# Plant callus-derived shikimic acid regenerates human skin through converting human dermal fibroblasts into multipotent skin-derived precursor cells

**DOI:** 10.1186/s13287-021-02409-3

**Published:** 2021-06-11

**Authors:** Yoo-Wook Kwon, Shin-Hyae Lee, Ah-Reum Kim, Beom Joon Kim, Won-Seok Park, Jin Hur, Hyunduk Jang, Han-Mo Yang, Hyun-Jai Cho, Hyo-Soo Kim

**Affiliations:** 1 Strategic Center of Cell and Bio Therapy for Heart, Diabetes & Cancer, Biomedical Research Institute, Seoul National University Hospital, Seoul, 03080 Republic of Korea; 2 Clinical Research Team, SK Chemical, Life Science Biz., Seongnam-si, Gyeonggi-do, 13494 Republic of Korea; 3 Skin Research Division, AMOREPACIFIC Corp. R&D Unit, Yongin, -si, Gyeonggi-do, Republic of Korea; 4 Departments of Dermatology, Chung-Ang University College of Medicine, Seoul, Republic of Korea; 5 Department of Convergence Medicine, Pusan National University School of Medicine, Yangsan, 50612 Korea; 6 Molecular Medicine and Biopharmaceutical Sciences, Graduate School of Convergence Science and Technology, Seoul National University, Seoul, 03080 Republic of Korea; 7 Cardiovascular Center & Department of Internal Medicine, Seoul National University Hospital, Seoul, Republic of Korea

**Keywords:** Shikimic acid, Cell reprogramming, Neural precursor, Skin-derived precursor, Single chemical-derived trans-differentiation

## Abstract

**Background:**

The human skin-derived precursors (SKPs) are a good cell source for regeneration. However, the isolation of SKP from human skin is limited. To overcome this drawback, we hypothesized that the component of plant stem cells could convert human fibroblasts to SKPs.

**Methods:**

Human dermal fibroblasts were treated with shikimic acid, a major component of *Sequoiadendron giganteum* callus extract. The characteristics of these reprogrammed cells were analyzed by qPCR, western blot, colony-forming assay, and immunofluorescence staining. Artificial human skin was used for CO_2_ laser-induced wound experiments. Human tissues were analyzed by immunohistochemistry.

**Results:**

The reprogrammed cells expressed nestin (a neural precursor-specific protein), fibronectin, and vimentin and could differentiate into the ectodermal and mesodermal lineage. Nestin expression was induced by shikimic acid through the mannose receptor and subsequent MYD88 activation, leading to P38 phosphorylation and then CREB binding to the nestin gene promoter. Finally, we confirmed that shikimic acid facilitated the healing of cut injury and enhanced dermal reconstruction in a human artificial skin model. Moreover, in a clinical study with healthy volunteers, plant callus extracts increased the expression of stem cell markers in the basal layer of the epidermis and collagen deposit in the dermis.

**Conclusions:**

These results indicate that shikimic acid is an effective agent for tissue regeneration.

**Supplementary Information:**

The online version contains supplementary material available at 10.1186/s13287-021-02409-3.

## Introduction

We have recently demonstrated that the protein extract from mouse embryonic stem cells (ESCs) or induced pluripotent stem cells (iPSCs) has a potential to convert mouse fibroblasts into iPSCs [1, 2]. Afterwards, we investigated whether human iPSCs could be generated by treatment with human ESC extract; however, we were unable to optimize the reprogramming method because it required a large amount of human ESC protein extract (20–50 mg). It was not possible to repeatedly obtain this amount per experiment because of the high expenditure of the culture system and batch-to-batch variations. To overcome these obstacles, we searched for a compound that could generate stem/progenitor cells with multi-potency and allow us to maintain them in an easy and inexpensive way. Based on recent findings, we hypothesized that plant callus extract could be the most appropriate candidate material for optimizing the process.

The formation of calluses in plants is induced by strong activation of the meristem [3], which hosts plant stem cells and is the ultimate source of all tissues in a plant. Calluses are massive growth of cells and accumulation of callose, a plant polysaccharide associated with wounds. It can be produced from single undifferentiated cells, which are totipotent, i.e., able to regenerate the whole body of a plant. In plants, regeneration is triggered by the loss of a tissue structure by injury, e.g., by excision of the tip of a stem, leaf, or root. In response, the plant callus acquires features similar to meristematic cells and develops into new stem cells that are capable of forming new individual plants [4]. Interestingly, the extract of plant calluses can be used for effective treatment of animal cells: a recent study showed that the extract of Swiss apple callus enhanced human stem cell proliferation, prevented apoptosis of umbilical cord blood stem cells from UV radiation, and reversed senescence in fibroblasts, leading to a prolonged lifespan of human skin cells [5].

The skin is a distinctive organ, because it has continuous cyclic cell turnover and is capable of regeneration to repair intrinsic and extrinsic injuries. This capacity normally originates from somatic tissue stem cells, such as mesenchymal stem cells in the epidermal basal layer, melanocyte stem cells, dermal stem cells, and hair follicles [6]. Dermal stem cells are responsible for maintaining homeostatic integrity and regeneration of the dermis. Skin-derived precursor cells (SKPCs) were successfully separated from adult skin in a trial to isolate neural precursor cells from the skin [7]. The characteristics of SKPCs are nearly identical to those of neural precursor cells (NPCs) [8]: they express not only vimentin, a type III intermediate filamentous protein that is expressed in mesenchymal cells, but also nestin, a type VI intermediate filament that is mostly expressed in the neural cell lineage. Moreover, SKPCs showed multipotency, namely differentiation into mesodermal and peripheral neural progeny [9]. They can be obtained from primary culture of skin biopsy samples from subjects, regardless of their age, site of biopsy, or disease [10]. It has recently been reported they can also differentiate into insulin-producing cells [11]. Since their first discovery, they have been extensively studied, and there are promising results regarding their use in regenerative and cell therapy. However, the isolation of SKPCs using the existing protocol that includes isolation from human skin has low efficiency and faces technical difficulties [12]. Therefore, we aimed to establish conditions for deriving SKPCs from human dermal fibroblasts (HDFs) that would overcome the obstacles the existing SKPC isolation protocol faces.

In this study, we demonstrated that shikimic acid, a major component of *Sequoiadendron giganteum* (SG) callus extract, could induce mesenchymal-to-epithelial transition (MET) and consequently convert human fibroblast to neural precursor-like skin precursor cells (NeuSKPCs). In addition, we identified that the receptor for shikimic acid is the mannose receptor and that it induced the major SKPC marker nestin through the MYD88-P38 MAPK-CREB pathway. Finally, the results of our artificial skin model and clinical study on aged female volunteers show that shikimic acid improves skin regeneration.

## Materials and methods

### Cell culture and plant callus preparation

Human dermal fibroblasts (Gibco) were cultured in high-glucose DMEM containing 10% fetal bovine serum, l-glutamine, and penicillin/streptomycin (all from Gibco).

### Colony-forming assay (stained with crystal violet)

Cells were washed twice in ice-cold PBS and fixed with ice-cold methanol for 10 min for permeabilization of cells. Expanded colonies were stained by 0.1% crystal violet (Sigma) and 10% ethanol in PBS solution for 5 min and PBS washed 4 times, and plate images were acquired. For quantification, the colonies were incubated with elution buffer (50% ethanol, 40% distilled water, 10% acetic acid) for 5 min and transferred to a 96-well plate; the OD at 580 nm was determined using a microplate reader.

### Generation of NeuSKPCs and immunocytochemistry

To generate NeuSKPCs, HDFs were seeded on an ultra-low attachment plate at 1.2 × 10^5^ cells/ml density. The composition of the NeuSKPCs media is DMEM:F12 (3:1) supplemented with 2% B-27 supplement (all from Gibco), 40 ng/ml basic fibroblast growth factor (R&D Biosystems), and 20 ng/ml EGF (Peprotech). The media were changed, and shikimic acid was added every other day.

For immunocytochemistry analysis, NeuSKPCs were collected in a conical tube and centrifuged for segmentation. NeuSKPCs were prepared on slide glass. Primary antibodies, anti-nestin (1:100, Sigma Aldrich), anti-fibronectin (1:100, Santa Cruz Biotech), and anti-vimentin (1:100, Santa Cruz Biotech), were diluted in antibody diluent solution (Invitrogen) and incubated at 4 °C, overnight. Appropriate secondary antibodies (donkey anti-rabbit Alexa Fluor 488 and anti-mouse Alexa Fluor 555, 1:200) were used. The nucleus was stained with DAPI (1:1000). Images were obtained using confocal microscopy (LSM 510 Meta, Carl Zeiss).

### Reverse transcription and quantitative reverse transcription PCR (qRT-PCR)

Total RNA was extracted using RNeasy mini kit (QIAGEN), and cDNA was prepared with ReverTraAce qPCR RT Master Mix (Toyobo). Quantitative reverse transcription PCR was performed using FastStart SYBR Green (Roche) and analyzed with Applied Biosystems 7500 Real-Time PCR System. Obtained data were normalized to 18s rRNA and calculated into 2^−ΔCt^ method. Primers were listed in Table s1.

### Western blot analysis

Total protein was isolated from cells with RIPA cell lysis buffer (Thermo Fisher), supplemented with a complete protease inhibitor cocktail (Genedepot) and 1 mM DTT. Lysates were incubated on ice for 30 min and vortexed every 10 min. Protein concentration was determined by BCA assay (Thermo Fisher). Proteins were separated on 8% gradient SDS-PAGE gels and transferred from gel onto the polyvinyl difluoride (PVDF) membrane. The membrane was blocked with 5% bovine serum albumin (BSA) and incubated overnight at 4 °C with primary antibodies. Detection was done using the Novex ECL HRP Chemiluminescent substrate reagent kit (Invitrogen). Western blot data were quantified by the ImageJ software.

### Microarray data acquisition and analysis

Hybridized microarrays were scanned using a DNA microarray scanner and quantified with the Feature Extraction Software (Agilent Technologies). All data normalization and selection of genes with a specific fold change were performed using human GE 4 X 44k (v2) Array (Agilent Technologies). All numerical data were normalized with intensity-dependent normalization (LOWESS), where the ratio was reduced to the residual of the Lowess fit of the intensity vs. ratio curve by using GeneSpring 7.3. >2× fold changed genes were selected and considered as differentially expressed genes (DEGs). Functional annotation of analyzed genes was performed according to the Gene Ontology Consortium. Gene classification was based on searches done using DAVID (http://david.abcc.ncifcrf.gov/).

### Tissue regeneration assay

The EpiDermFT-400 in vitro full-thickness skin tissue model was purchased from MatTek Corporation (Ashland). The system consists of human-derived epidermal keratinocytes (NHEK) and human-derived dermal fibroblasts (NHDF) which have been grown on a semipermeable membrane to form a multilayered, highly differentiated model of the human dermis and epidermis. The tissues were cultured in a 6-well plate at 37 °C, 5% CO_2_ using a serum-free medium, according to the manufacturer’s instruction. Upon arrival, 24 plugs were immediately transferred to a 6-well plate with 2 ml EFT-400 medium and incubated overnight at 37 °C, 5% CO_2_. After overnight equilibration, the medium was replaced with a pre-warmed fresh supply. The EpiDermFT-400 tissue was wounded by CO_2_ laser (100 mJ, 200 spots/cm^2^, 300 μm spot diameter, eCO_2_ Mosaic Fractional Laser, Lutronic Co., South Korea), cultured in ETF-400 culture media containing shikimic acid (2 mM, 20 mM) or control culture media for 4 days. After treatment, EFT-400 tissues were fixed in 10% neutral-buffered formalin, embedded in paraffin, sectioned 8 mm thickness.

### Clinical study design

Health adult volunteers who have wrinkle or sag on inner arms (age > 50, *n* = 8) were recruited for the study in the Chung-Ang University Hospital (Seoul, South Korea). Prospective volunteers who had any systemic or skin diseases or were using any medications for cosmetic purposes in the last 1 month were excluded. Those who had taken any systemic or topical medications 1 month or 2 weeks prior to enrollment, respectively, were also excluded. We randomly assigned the volunteers to one of the following two groups (*n* = 4) by using double-blind methods: *SG* callus 1.8% (shikimic acid; 36.25 mM) complying cosmetic cream treated; vehicle cosmetic cream treated. During the study period, the use of systemic medications and topical agents, or washing of the test sites, was prohibited. On the first day, 2 sites were marked on the inner arms of volunteers, before applying topical sample. The whole punch skin biopsy (3 mm in diameter) was taken from the left inner arm, as a naive normal control on the assumption that each inner arm’s tissue histology is the same. The naive normal control tissues obtained from the left inner arms were immediately embedded in optimal cutting temperature (OCT) compound, frozen in liquid nitrogen, and stored at −80 °C until the preparation of frozen sections or placed in 10% phosphate-buffered formalin. These naive normal control tissues were used negative controls and were stained concurrently to validate the staining procedure.

After the preliminary inspection, we applied creams (0.2 ml) on each inner arm of volunteers by using an occlusive patch for 3 weeks. After 3 weeks, the whole punch skin biopsy (3 mm in diameter) was taken from the right inner arm. The same tissue treatment process was performed.

After all of the tissue samples (normal control tissues and treated tissues) were obtained, skin specimens were prepared for immunohistochemical staining at the same time and imaged with the same settings. Five-micron cryostat sections were cut on a cryostat microtome and then fixed in acetone for 15 min. After three washes in phosphate-buffered saline (PBS), the sections were incubated in methanol with 0.3% hydrogen peroxide for 10 min to block the endogenous peroxidase activity. Nonspecific antibody binding was blocked by incubating the sections with 10% normal donkey serum. Immunostaining was performed for detecting stemness markers of the epidermal layer by using anti-β1 integrin and anti-α6 integrin antibodies (Santa Cruz Biotech). The stained tissue sections were photographed using a Zeiss microscope (Axioskop 2), and images were obtained with a digital camera (Olympus, Japan).

### Statistical analysis

All experiments and groups were at least triplicates, and all data were calculated as mean ± standard deviations (SD). Group comparisons were performed by Student’s t test when compared to two groups and AVOVA statistic test when compared more than that. *P* values ≤0.01 were considered as statistically significant values. All statistical analyses were conducted using the GraphPad Prism software.

## Results

### Shikimic acid from plant stem cell extract induces dedifferentiation of human somatic cells

**Fig. 1:**
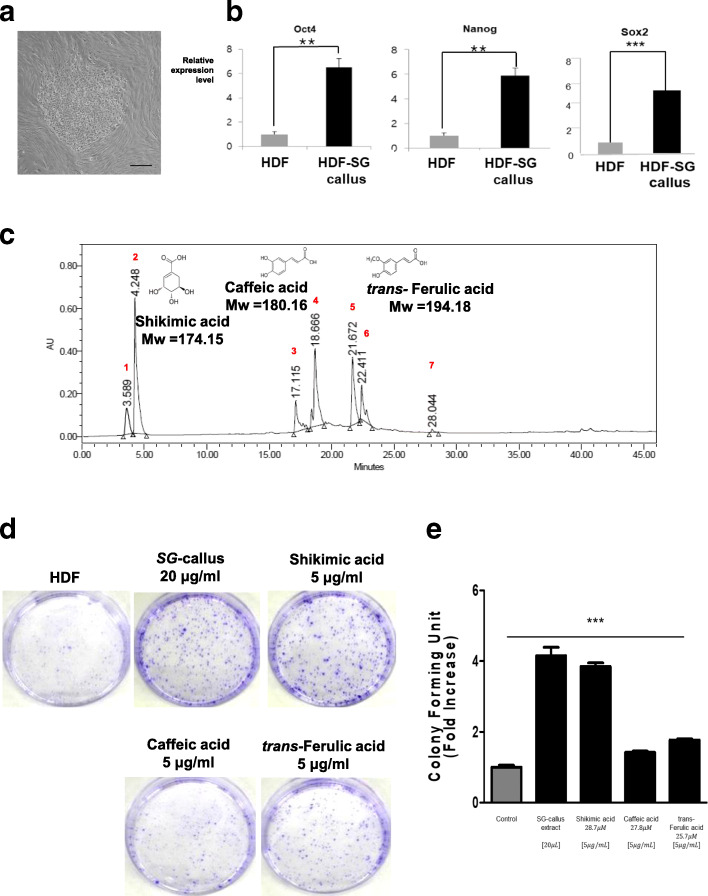
*SG*-callus extract and its major component, shikimic acid, induce stemness in human fibroblast and expression of pluripotent stem cell markers. **a** Bright-field images of HDF after treatment with SG callus extract (500 μg/ml) for 7 days. Scale bar = 500 μm. **b** RNA expression of OCT4, NANOG, and Sox2 were analyzed by qRT-PCR. It was normalized to 18s RNA (*N* = 3 in each group, **p < 0.01). **c** HPLC chromatogram of the SG-callus extract detected at the UV wavelength of 230 nm. The peak (RT = 4.248 min) is assigned to the shikimic acid. The peak (RT = 18.666 min) is assigned to the caffeic acid. The peak (RT = 21.672 min) is assigned to the trans-ferulic acid. **d** A colony formation assay was performed on naive HDF and HDF treated with SG-callus extract or shikimic acid, caffeic acid, and trans-ferulic acid at 10 days after treatment. Scale bar = 500 μm. Representative pictures are shown. **e** Relative stained colonies are quantified (*N* = 3 in each group, *p < 0.05, ***p < 0.001 compared with the control)

To investigate whether plant stem cell extract could reprogram somatic cells, we treated HDFs with SG callus extract. Interestingly, the cell morphology changed after 7 days of treatment (Fig. 1a). This suggested that mesenchymal-to-epithelial transition (MET) had occurred, which is an early event in the reprogramming of somatic fibroblasts into pluripotent stem cells. Moreover, after exposure to the extract, the HDFs showed increased expression of stemness genes, such as OCT4, NANOG, and Sox2 (Fig. 1b). These findings suggested that the extract could reprogram human skin cells to an undifferentiated state, such as multipotent precursor cells. To identify the main active components of the SG callus extract, we performed HPLC analysis and identified three major compounds (Fig. 1c): shikimic acid, caffeic acid, and trans-ferulic acid. Since there are no previous reports on the reprogramming effects of any of the compounds, we first aimed to compare each compound’s potential to induce reprogramming. We treated the HDFs with 5 μg/mL of each compound separately and cultured them for 10 days. Only shikimic acid distinctly upregulated alkaline phosphatase (AP) activity similar to SG callus extract (Fig. 1d, e), and significantly increased colony formation by 4-folds (*p* < 0.001; Fig. 1f). On the other hand, caffeic and trans-ferulic acid showed little reprogramming potency (Fig. 1d–f). However, while these data indicated that shikimic acid could convert somatic cells into pluripotent cells, we could not observe completely mature iPSCs after a single treatment with it (Figure S1); thus, it can be said that shikimic acid might act by increasing the level of stemness of somatic cells.

### Shikimic acid converted HDF spheres into neural precursor-like skin precursor cells (NeuSKPCs)

**Fig. 2:**
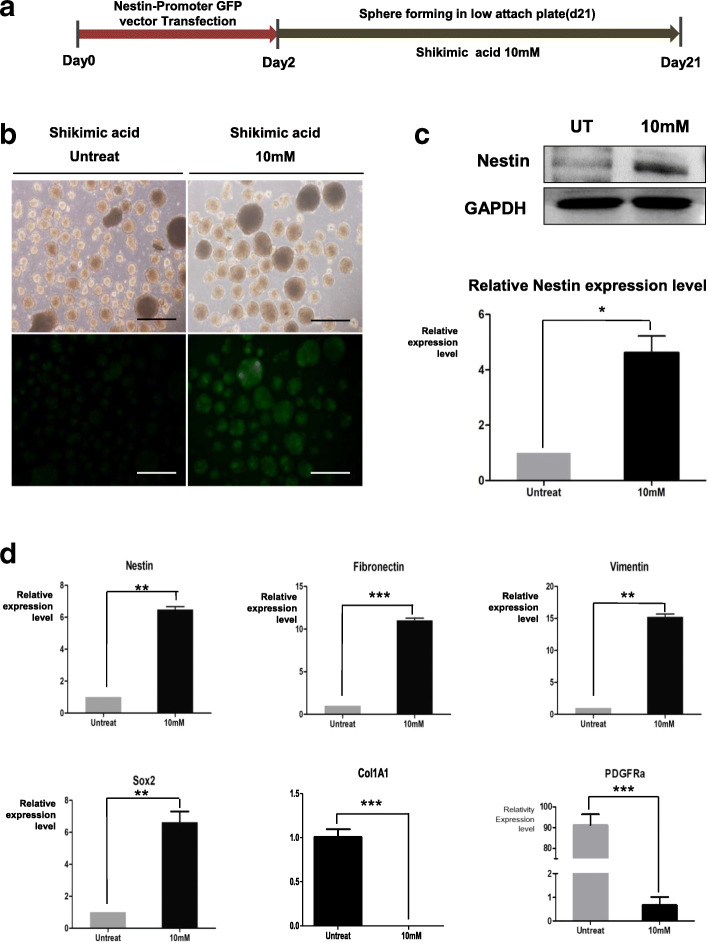
NeuSKPCs are induced by treatment of shikimic acid. **a** Scheme of generation of NeuSKPCs. **b** Morphology of NeuSKPCs. Nestin promoter GFP was activated in the shikimic acid-treated groups (scale bar = 500 μm). **c** Western blot analysis was conducted using an anti-nestin antibody (upper panel) in shikimic acid untreated or treated spheroids, and the quantified result is shown in the lower panel (*p < 0.05). **d** qRT-PCR analysis of SKP-associated genes, nestin, fibronectin, vimentin, pluripotency-associated gene, Sox2, fibroblast-specific genes, Col1A1, and PDGFRa. It was normalized to 18s RNA (*N* = 3 in each group, **p < 0.01, ***p < 0.001 compared with the control)

To investigate whether nestin, the neural precursor cell marker, is expressed in shikimic acid-treated HDF spheres, we transfected HDFs with a nestin promoter-GFP fusion vector as a reporter. GFP expression increased depending on the dose of shikimic acid (1–10 mM). We found that the optimal concentration of shikimic acid for enhancing nestin-GFP promoter activity was 10 mM (Figure S2A). Figure 2a shows a scheme of NeuSKPCs induction. The transfected HDFs were seeded on an ultra-low attachment plate; spheres were generated on day 3 and maintained for 3 weeks. GFP was observed in shikimic acid-treated spheres from day 4 (Fig. 2b). Compared to the untreated spheres, shikimic acid-treated spheres showed enhanced mRNA and protein expression of nestin, vimentin, and fibronectin, which are known as SKPC markers. Interestingly, the expression of fibroblast-specific genes (Col1A1 and PDGFRa) were decreased (Fig. 2c, d). These data suggest that shikimic acid converted fibroblasts into NeuSKPCs from the early stage of sphere formation.

### Characterization of shikimic acid-induced NeuSKPCs: colony-forming ability and telomere elongation

**Fig. 3:**
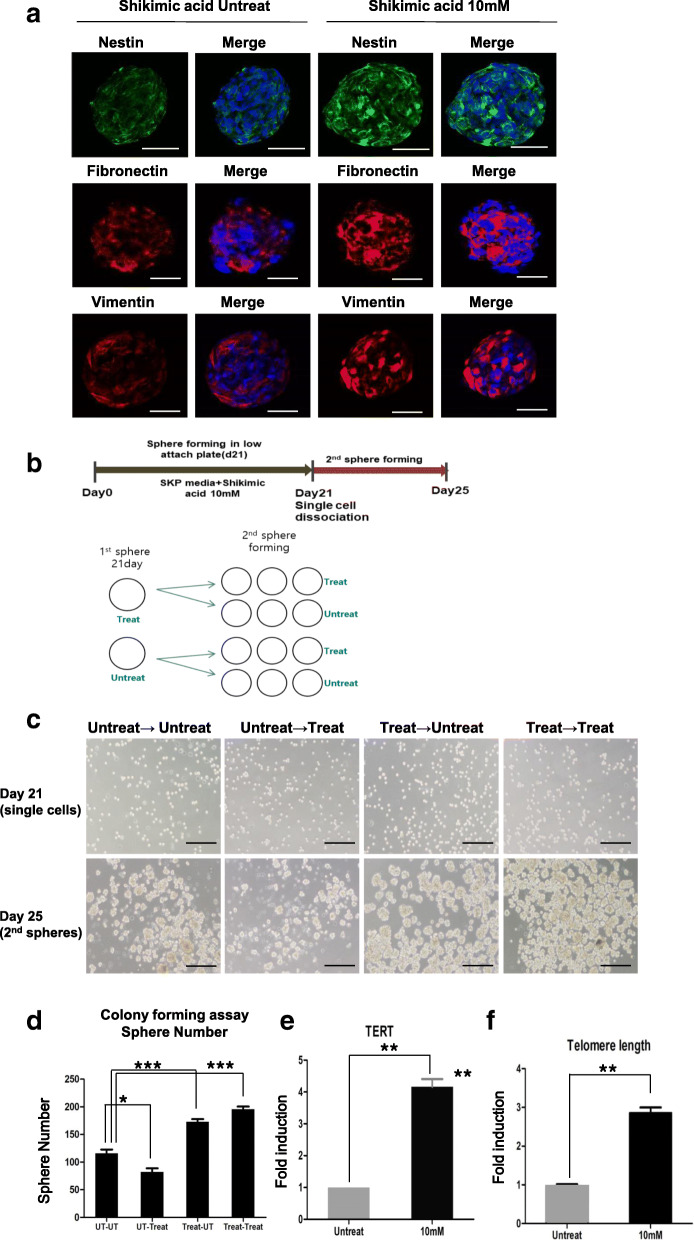
Characterization of shikimic acid-induced NeuSKPCs. **a** Immunofluorescence of SKP markers including anti-nestin (green), anti-fibronectin, and anti-vimentin (red) in shikimic acid-induced NeuSKP on day 21. DAPI was used to identify the nucleus of the cells (scale bar = 50 μm). **b** Scheme of colony-forming assay (secondary sphere formation). **c** Bright-field images of secondary NeuSKPCs with or without shikimic acid for 4 days (scale bar = 500 μm). **d** The number of secondary spheres from matured NeuSKPCs (*N* = 5 in each group, *p < 0.05, ***p < 0.001). **e** qRT-PCR analysis of TERT in matured NeuSKPCs (10 mM shikimic acid was treated for 21 days, **p < 0.01 compared with the untreated control). **f** Comparison of the average telomere length ration between shikimic acid untreated or treated (**p < 0.01 compared with the untreated control)

The optimized time point for sphere maturation is 3 weeks. The shikimic acid-treated cells showed higher expression levels of nestin, fibronectin, and vimentin than the untreated cells (Fig. 3a, Figure S3A).

To evaluate the self-renewal ability of NeuSKPCs, we performed colony-forming assays. First, we dissociated matured primary spheroids into single cells in order to assess secondary sphere formation. The single cells were seeded on ultra-low attachment plates to form secondary spheres with or without shikimic acid treatment (Fig. 3b). The treated group generated an approximately 2-fold greater number of secondary spheres than the untreated group. Interestingly, even after treating the single cells that were not treated with shikimic acid during the generation of primary spheres (UT-Treat), the number of secondary spheres did not increase (Fig. 3c, d). These data suggest that shikimic acid could act by working on the early stage of induction of NeuSKPCs.

To investigate the mechanism of how shikimic acid could convert fibroblast to NeuSKPCs, we performed global gene expression analysis, comparing human dermal fibroblast with or without shikimic acid. RNA samples were labeled with Cy3 (HDF) and Cy5 (shikimic acid-treated HDF) then analyzed on Agilent human GE 4 X 44 k (v2) Array. Scatterplots demonstrated that shikimic acid could change the global gene expression pattern. In detail, the profile of differentially expressed genes (DEGs) selected by a fold changes more than 2 revealed that 6348 genes were differently expressed after shikimic acid treatment (Figure S4A). Since the morphology of HDF dramatically changed epitheloid by treatment of shikimic acid (Figure S1B) and this mesenchymal-to-epithelial transition (MET) phenomenon is one of the first noticeable changes during reprogramming [13], we analyzed the genes regulating MET from the profile of DEGs. Shikimic acid reduced the expression of mesenchymal cell markers such as Twist, Zeb1, and Zeb2, while it increased the expression of epithelial cell markers such as Claudin, Occludin, CRB3, and E-cadherin (Figure S4B). We confirmed this gene expression pattern using qRT-PCR (Figure S4C). As we expected, MET-associated genes were noticeably changed (Figure S4C). The other interesting reprogramming-related gene is TERT. As it is reported that telomeres are elongated during reprogramming [14], we investigated whether shikimic acid treatment affects the telomere length. Indeed, TERT, a major component of telomerase known to elongate telomeres of stem cells [15], was highly expressed, and telomeres were elongated after shikimic acid treatment (Fig. 3e, f).

### NeuSKPCs induced by shikimic acid can differentiate into the mesodermal and ectodermal lineage

**Fig. 4:**
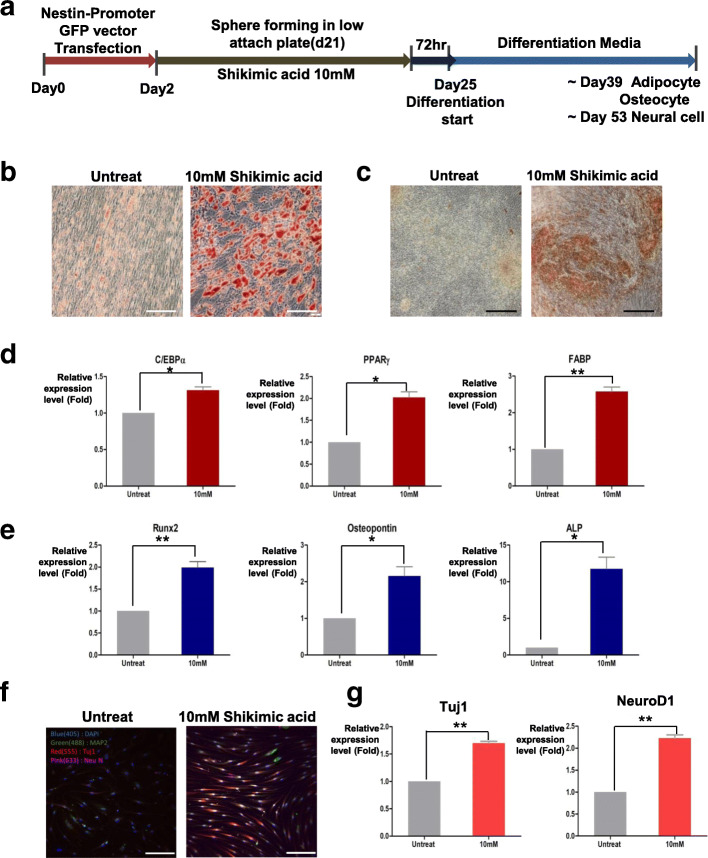
Shikimic acid-induced NeuSKPCs can differentiate into ectodermal and mesodermal lineage. **a** Scheme of differentiation for NeuSKPCs. **b** Lipid drops of adipogenic differentiated cells were stained by Oil red O (upper panel, scale bar = 100 μm). **c** Accumulated calcium of osteogenically differentiated cells were stained by von Kossa staining (scale bar = 200 μm). **d** qRT-PCR analysis of adipocyte-specific genes, CEBP, PPAR, and FABP (*N* = 3 in each group; ns, not significant, *p < 0.05, **p < 0.01). **e** qRT-PCR analysis of osteocyte-specific genes, Runx2, osteopontin, and ALP (*N* = 3 in each group, *p < 0.05, **p < 0.01). **f** Differentiated neural cells were stained with MAP2 (green), Tuj1(red), and NeuN (magenta) (scale bar = 100 μm). **g** qRT-PCR analysis of neuron-specific genes, Tuj1, and NeuroD1 (*N* = 3 in each group, **p < 0.01)

The differentiation potential of SKPCs was reported to be restricted to the mesodermal and ectodermal lineages [9]. To evaluate whether the NeuSKPCs induced by shikimic acid are multipotent, we induced their differentiation into mesodermal and ectodermal lineage cells by plating them onto a gelatin-coated dish in the presence of adipogenic, osteogenic, and neuronal differentiation media (Fig. 4a) [16]: their morphology changed depending on the media used. The cells were positive for Oil Red O and von Kossa staining (Fig. 4b, c) and expressed the adipocyte-specific markers C/EBPα, PPARγ, and FABP (Fig. 4d) and osteocyte-specific markers RUNX2, osteocalcin, and ALP (Fig. 4e). When nestin promoter-GFP-positive spheres were attached to the plate, the cells began to extend from the center of the sphere, and the fluorescence of GFP was sustained. As differentiation progressed, the nestin promoter became inactivated, and no GFP expression was observed on differentiation day 2. As the cells differentiated into the neural cell lineage (differentiation day 7), the nestin promoter was reactivated and the GFP signal reappeared (Figure S5A, B). The differentiated neural cells expressed MAP2, Tuj1, NeuN, and NEUROD1 (Fig. 4f, g); these data corresponded to the characteristics of SKPCs [9].

### Shikimic acid-induced nestin expression via the MYD88/MAPK/CREB signaling pathway

**Fig. 5:**
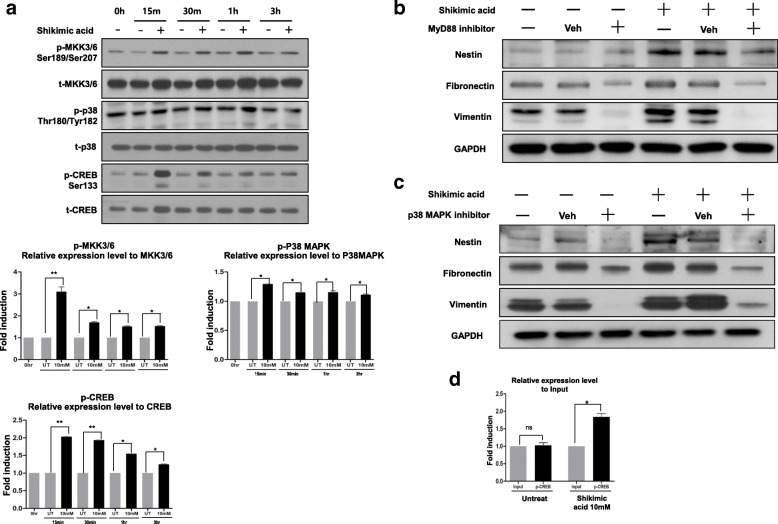
Shikimic activates nestin expression via the MyD88/p38/CREB signaling pathway. **a** After the designated incubation time with shikimic acid, the phosphorylation level of MAPK-kinase (MKK)3/6, p38 MAPK, and CREB was determined (upper). Quantification of the obtained results (*N* = 3 in each group, *p < 0.05, **p < 0.01, ***p < 0.001 compared with the control) (lower). **b** Western blot analysis of nestin, fibronectin, and vimentin after treatment of MyD88 inhibitor and shikimic acid. **c** Western blot analysis of nestin, fibronectin, and vimentin after treatment of p38 inhibitor and shikimic acid. **d** Chromatin immunoprecipitation (ChIP) assay on the promoter of nestin. Phosphor-CREB antibody was used for the ChIP assay (*N* = 3 in each group, *p < 0.05)

We investigated the molecular mechanism by which shikimic acid induced the expression of nestin or converted fibroblast to neural precursor cell. Shikimic acid reportedly interacts with the cell surface mannose receptor (MR) [17] and activates MYD88 through TLR2 [18]. Downstream of MYD88, the signaling diverges into three MAPK pathways, MAPK-Kinase (MKK)1/2, MKK3/6, and MKK7 [19]. MKK1/2 regulates ERK1/2 activity, while MKK3/6 and MKK7 regulate the phosphorylation of P38 MAPKs and JNK, respectively [20, 21]. We thus investigated the phosphorylation of these molecules after shikimic acid treatment. After 15 min of shikimic acid treatment, MKK3/6, P38, and CREB were phosphorylated (Fig. 5a). On the other hand, there was no change in the phosphorylation level of MKK1/2, and phospho-ERK1/2 was downregulated. There was no significant change in the phosphorylation levels of MKK7 and JNK (Figure S6). These data suggested that shikimic acid activates MKK3/6 phosphorylation, which led to the phosphorylation of downstream signals, including P38 MAPKs and CREB. To confirm whether MYD88 and P38 are required for the induction of nestin by shikimic acid, we assessed nestin expression following the addition of signal inhibitors to the culture medium. HDFs were seeded on ultra-low attachment plates and maintained for 4 days with or without shikimic acid, and the concentration of the inhibitors was optimized (Figure S7). After treatment with the MYD88 inhibitor ST2825, nestin expression considerably decreased (Fig. 5b, Figure S8A). The inhibitor of P38 MAPK activity, SB203580, inhibited shikimic acid-induced nestin expression (Fig. 5c, Figure S8B). On the other hand, the JNK inhibitor, SP600125, did not affect nestin expression (data not shown). Therefore, our results indicated that shikimic acid specifically activated the MYD88-MKK3/6 signaling axis, which phosphorylated P38 MAPKs and CREB. To investigate whether the transcription factor CREB binds to the nestin promoter, we performed ChIP assays and found that the nestin promoter recruited CREB (Fig. 5d). After shikimic acid treatment, the binding of CREB increased 2-fold compared with the untreated control, indicating that CREB bound directly to the nestin promoter and increased the expression of nestin after shikimic acid treatment.

### Shikimic acid or plant callus extracts enhanced human skin regeneration in the artificial skin model and in the clinical study with healthy volunteers

**Fig. 6:**
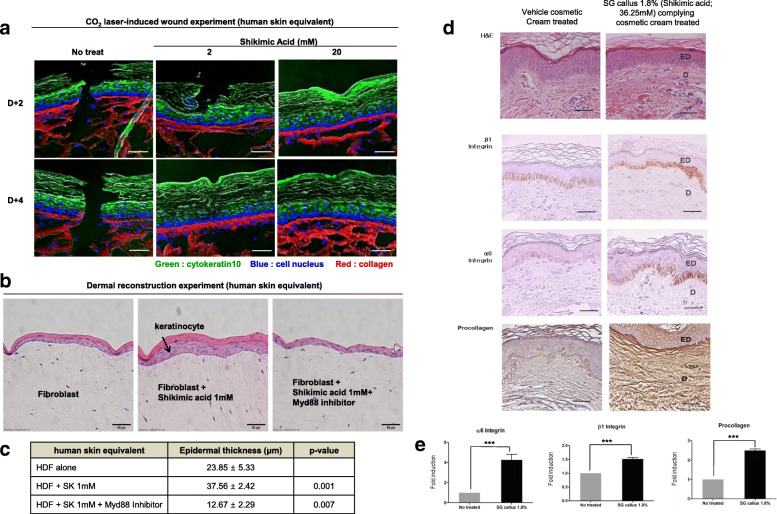
Shikimic acid facilitates wound healing and enhances dermal reconstruction in the artificial human skin ex-vivo model. **a** Representative fluorescent immuno-histologic staining of cytokeratin (green) in the epidermis and cell nucleus (blue) in the basement membrane and collagen (red) in the dermis from human skin equivalent tissue. Tissue was frozen in OCT and stained by the immunofluorescence method. Data are representative of *N* = 3. Scale bar = 10 μm. **b** The dermal layers were seeded with 3 different fibroblasts: Non-treated fibroblast (control), fibroblast treated with shikimic acid, or fibroblast treated with shikimic acid plus MyD88 inhibitor. Keratinocytes were seeded over the dermal layer. This skin equivalent was cultured in the air-liquid interface for 8 days. On 8 days, the paraffin sections were stained for hematoxylin and eosin (scale bar = 50 μm). **c** The quantification of epidermal thickness (*N* = 3 in each group compared with control HDF, *p* < 0.001 or *p* < 0.007). **d** *SG*-callus extract (36.25 mM shikimic acid) increased the expression level of β1 integrin and α6 integrin that are stem cell markers at the basal layer of the epidermis. It also increased collagen amounts in the dermis (brown color in immunostaining) (scale bar = 100 μm). **e** *N* = 8, treated group compared with vehicle cream treated group, ***p < 0.001. ED, epidermis; D, dermis

To test the effect of shikimic acid on skin regeneration, we used two different models, such as ex vivo human skin equivalent tissue model and in vivo clinical study with female volunteers. Firstly, we prepared a wounded skin equivalent tissue model and treated it with 2 or 20 mM shikimic acid. Since we used a fixed laser parameter of 100 mJ, 200 spots/cm^2^ 300 μm spot diameter to wound the skin, it was presumed that the injury sizes of all skin equivalent tissue models were the same at the baseline. Two days after inducing the wound using a CO_2_ laser, the epidermal and dermal parts were repaired in shikimic acid-treated skin, but not in the untreated control. Four days after wound induction, complete re-epithelialization and restoration of the dermis occurred in the shikimic acid-treated tissue, but not in the untreated control. Specifically, the collagen structure in the dermal layer became denser in the treated skin than in the untreated control (Fig. 6a). Secondly, to investigate the dermal reconstruction potential of shikimic acid, we prepared a dermal equivalent that contained human epidermal neonatal keratinocytes on the dermal layer and human dermal fibroblasts under the dermal layer and treated it with shikimic acid. The treatment significantly increased the proliferation of keratinocytes compared with the control, and this effect was abolished by MYD88 inhibitor treatment (Fig. 6b, c). These data suggest that, via the MYD88 pathway, shikimic acid can stimulate fibroblasts under the dermal layer to secrete cytokines, leading to the proliferation of keratinocytes.

Finally, to confirm in healthy female volunteers whether the SG callus extract containing shikimic acid could increase skin tissue stemness markers and enhance the composition of the extracellular matrix of human skin, we performed a clinical study with healthy female volunteers, aged over 50 years (*n* = 8). The 1.8% SG callus cream (36.25 mM shikimic acid) or vehicle cream was applied topically using occlusive patches for 3 weeks. Two representative immuno-histologic images were prepared from the biopsied skin tissue of the inner arms of the treated patients (vehicle cream-treated skin vs 1.8% SG callus cream-treated skin). In hematoxylin and eosin staining, all samples showed an upper stratified squamous epithelium, a dense, fine fibrous dermis, and a basement membrane between the epidermis and dermis. There was no significant change in epidermal morphology (Fig. 6d). In the SG callus-treated skin, however, the expression of skin and neural stem cell markers (β1 and α6 integrin) were increased in the epidermal basal layer, and the procollagen expression level was increased in the dermis compared to the vehicle cosmetic cream treated skin (Fig. 6d, e). These data demonstrate that shikimic acid enhances skin remodeling by inducing stemness markers and enhancing the composition of the extracellular matrix of the skin.

## Discussion

### Plant stem cells extract converts human fibroblast to skin precursor cells

In plants, nuclear reprogramming occurs without manipulation of the nuclear status by external triggers [4, 22]. We predicted that plant stem cells, which possess the ability to convert mature somatic cells to immature blastemal cells in plants [23], might have the capacity to reverse the fate of human somatic cells. Indeed, after 10 days of callus extract treatment, the HDFs in our study changed morphologically, resembling epithelial cells (Figure S1), and expressed stemness markers including Oct4, Sox2, Klf4, and Nanog (Fig. 1b and Figure S9). However, we could not observe completely mature iPSCs after a single treatment with it (Fig. 1a). This mechanism remained to be investigated, but we know that one of the major iPSC generating factors, c-Myc, was not induced by callus extract. Since C-Myc was a very important boosting factor during iPSC generation, we assumed that a single treatment of callus extract did not induce pluripotent stem cells.

We demonstrated that, of the three major compounds of the callus extract, shikimic acid was the one that could induce reprogramming. We then investigated whether the reprogrammed cells could differentiate into different types of cells and whether they possessed self-renewal capacity. Although a single treatment with shikimic acid failed to generate complete iPSCs (Figure S1B), it did induce transition into SKPCs that exhibited stemness and could differentiate into the mesodermal and ectodermal lineage (Fig. 4). These results suggest that a single molecule could convert somatic cells into NeuSKPCs. In addition, we confirmed the self-renewal ability of NeuSKPCs: single cells originating from dissociated NeuSKPCs could generate secondary spheres, and shikimic acid-treated cells could generate an increased number of spheres expressing markers of NeuSKPCs (Fig. 3a–c). Moreover, the self-renewal ability of shikimic acid affected telomeres by increasing the expression of TERT, leading to telomere elongation (Fig. 3d–f). These data suggest that shikimic acid can convert somatic to precursor cells.

### Shikimic acid acts through the mannose receptor and the MYD88/P38/CREB signaling pathway

Shikimic acid is an important intermediate in the biosynthesis of lignin and most alkaloids of plants. It has been found in many organs, such as stems, leaves, and fruits, in a variety of plants [24]. Industrially, it is generally utilized as a starting material for the synthesis of oseltamivir, a drug against the influenza virus [25]. Although there are numerous reports about chemical and pharmaceutical applications of shikimic acid, this is the first report on applying shikimic acid to cell reprogramming and regeneration.

We elucidated the biological mechanism of shikimic acid-driven cell fate conversion, namely, shikimic acid is known to interact with C-type lectin-like domains (CTLD) of the mannose receptor, which leads to subsequent activation of the MYD88 signaling pathway. The mannose receptor selectively recognizes mannose, *N*-acetyl glucosamine, and fructose residues. Thus, shikimic acid could act as a mimic of these carbohydrates [16]. The other major components of the SG callus extract, caffeic acid and trans-ferulic acid, are not recognized by the mannose receptor, making shikimic acid the only SG callus compound that can induce reprogramming. In particular, shikimic acid activates the MKK3/6-P38 MAPK-CREB pathway, which activates the promoter of nestin (Fig. 5). Since nestin is a key molecule in SKPC generation, increasing its expression is an important event in reprogramming. In fact, MYD88 downstream signaling molecules JNK, P38, and ERK are known to affect reprogramming [19, 26, 27]. Among these, shikimic acid specifically activated the P38-CREB signaling axis via the phosphorylation of MKK3/6. Moreover, we confirmed that a CREB-biding sequence exists in the nestin promoter (Fig. 5d). Furthermore, a CREB-binding sequence was also found in the fibronectin and vimentin promoters, so there is a possibility that they share the MKK3/6–P38–CREB signaling with nestin. However, future studies should precisely determine the activation mechanism of fibronectin and vimentin.

### Shikimic acid enhances human skin regeneration

Human skin equivalents are bioengineered substitutes composed of primary human skin cells (keratinocytes, fibroblasts, and/or stem cells) and extracellular matrix components (mainly collagen). They are commercially used as clinical skin substitutes and as models for permeation and toxicity screening. In this study, we used such a model to test whether shikimic acid could enhance skin regeneration. We observed complete repair of the dermis after treatment with shikimic acid. Complete re-epithelialization and restoration of the dermis were observed, accompanied with a denser collagen structure (Fig. 6a). According to these results, shikimic acid not only affects dermal fibroblast fate by increasing the level of stemness, but also improves wound healing.

Moreover, we clinically tested the topical application of the SG callus extract and found that the levels of beta1 and alpha6 integrin and procollagen were increased in the treated dermis (Fig. 6d). Beta1 integrin has been known as a major molecule to regulate neural stem cell (NSC) growth factor responsiveness. It may provide NSC with the capacity to react to a dynamic “niche” and to respond adequately by either remaining as stem cells [28]. Integrin alpha6 are also broadly expressed among different types of stem cells and play an important role in maintaining self-renewal of stem cells [29]. Since integrin-linked kinase (ILK) regulates the niche of epidermal stem cell in hair follicle [30], shikimic acid-induced integrin may affect hair regeneration.

These data demonstrated that shikimic acid can enhance skin remodeling by inducing a “niche” of stem cells in the skin. Therefore, we expect that shikimic acid can be used as a novel material in skin regeneration bio-therapeutics or cosmetics. Although we have shown the potential effect of shikimic acid-containing cosmetic cream in this preliminary clinical study, the ability of the active ingredient in penetrating the epidermal barrier must be investigated to be used in cosmetics. Therefore, based on the results of this study, further studies are needed to investigate how and how much of the active ingredient crosses the epidermal barrier in vivo in order to exert a substantial effect on the dermis. Last but not least, we also anticipate that the efficiency of iPSC generation can be improved by simultaneous treatment with Yamanaka factors and shikimic acid since it was able to induce MET, cell proliferation, self-renewal, and stemness markers, which are essential factors of cell reprogramming.

In conclusion, by demonstrating that shikimic acid, a major chemical compound of plant callus, could induce fate conversion from a somatic cell to a skin precursor cell. We believe that our study made a considerable contribution in the field of reprogramming by discovering a novel compound that could induce the reprogramming process on human somatic cells.

## Supplementary Information

**Additional file 1: Figure S1-S9**.

**Additional file 2: Table S1-2**.

**Additional file 3.**

## Data Availability

The datasets used and/or analyzed during the current study are available from the corresponding author on reasonable request.

## Abbreviations

ESC: Embryonic stem cells
iPSCs: Induced pluripotent stem cells
HDF: Human dermal fibroblast
SKPCs: Skin-derived precursor cells
NPCs: Neural precursor cells
MET: Mesenchymal-to-epithelial transition
NeuSKPCs: Neural precursor-like skin precursor cells
ChIP: Chromatin immunoprecipitation
qPCR: Quantitative real-time polymerase chain reaction
C/EBP: CCAAT-enhancer-binding protein
PPAR: Peroxisome proliferator-activated receptor
ALP: Alkaline phosphatase
GFP: Green fluorescent protein
CREB: cAMP response element-binding protein
CTLD: C-type lectin-like domains
