# Supplementary material for: Plant callus-derived shikimic acid regenerates human skin through converting human dermal fibroblasts into multipotent skin-derived precursor cells
Source: Stem Cell Res Ther. 2021 Jun 11;12:346. doi: 10.1186/s13287-021-02409-3 (PMC8196440; doi:10.1186/s13287-021-02409-3)
Supplement: Supplementary file 1 — Additional file 1: Figure S1-S9. [file 13287_2021_2409_MOESM1_ESM.pptx]

## Slide 1
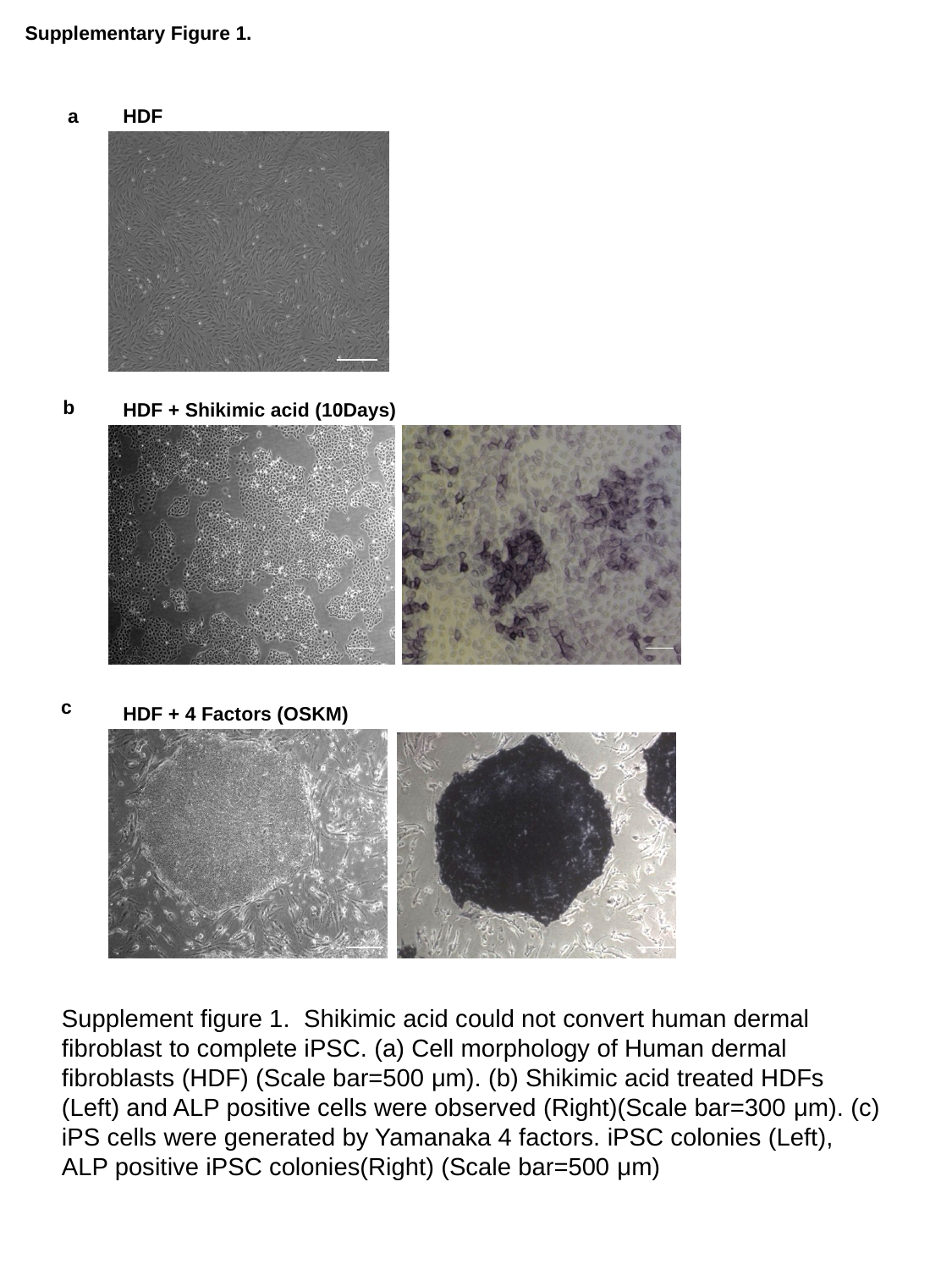

Supplementary Figure 1.
a
HDF
b
HDF + Shikimic acid (10Days)
c
HDF + 4 Factors (OSKM)
Supplement figure 1. Shikimic acid could not convert human dermal fibroblast to complete iPSC. (a) Cell morphology of Human dermal fibroblasts (HDF) (Scale bar=500 μm). (b) Shikimic acid treated HDFs (Left) and ALP positive cells were observed (Right)(Scale bar=300 μm). (c) iPS cells were generated by Yamanaka 4 factors. iPSC colonies (Left), ALP positive iPSC colonies(Right) (Scale bar=500 μm)

## Slide 2
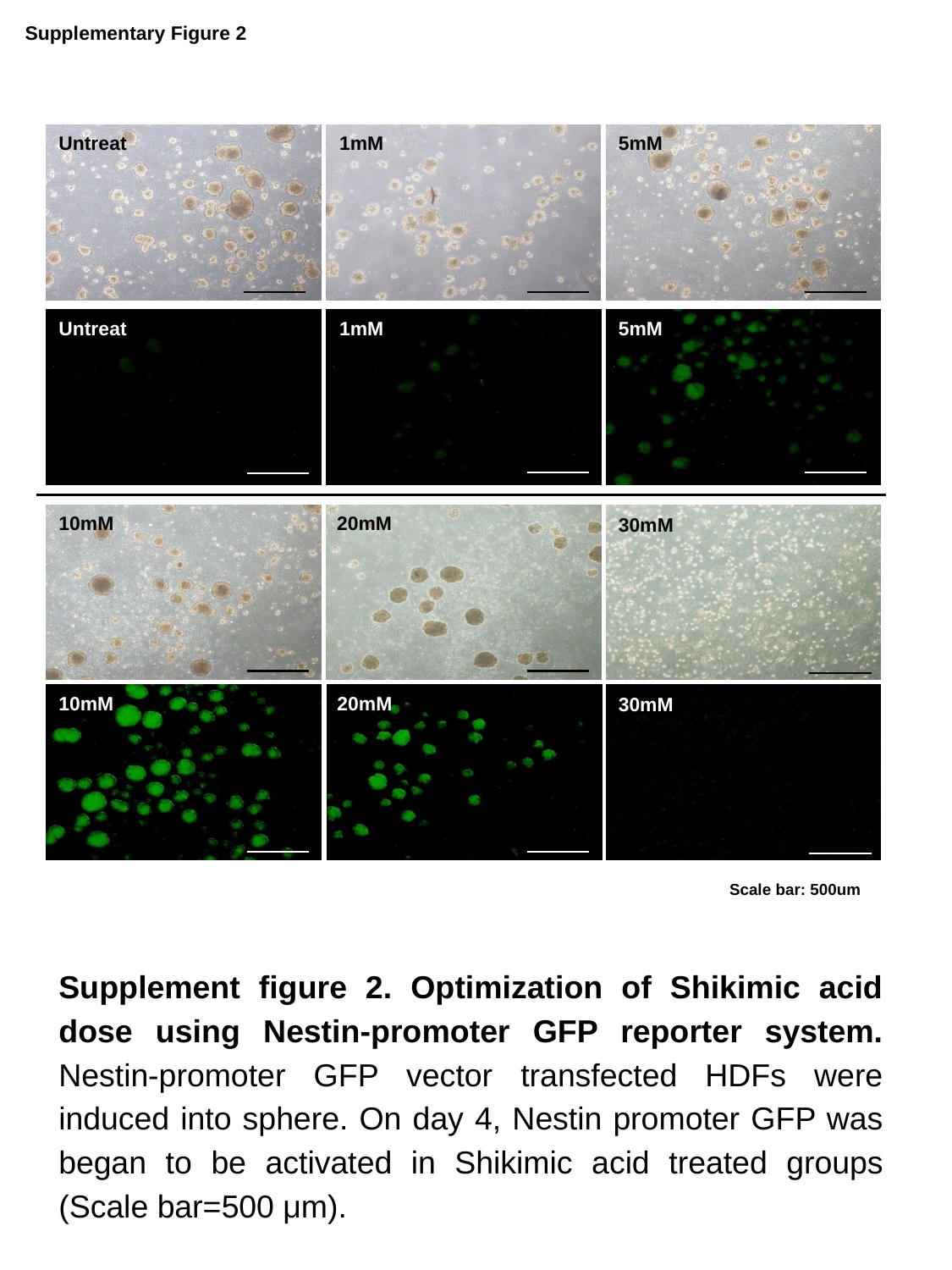

Supplementary Figure 2
Untreat
1mM
5mM
Untreat
1mM
5mM
10mM
20mM
30mM
10mM
20mM
30mM
Scale bar: 500um
Supplement figure 2. Optimization of Shikimic acid dose using Nestin-promoter GFP reporter system. Nestin-promoter GFP vector transfected HDFs were induced into sphere. On day 4, Nestin promoter GFP was began to be activated in Shikimic acid treated groups (Scale bar=500 μm).

## Slide 3
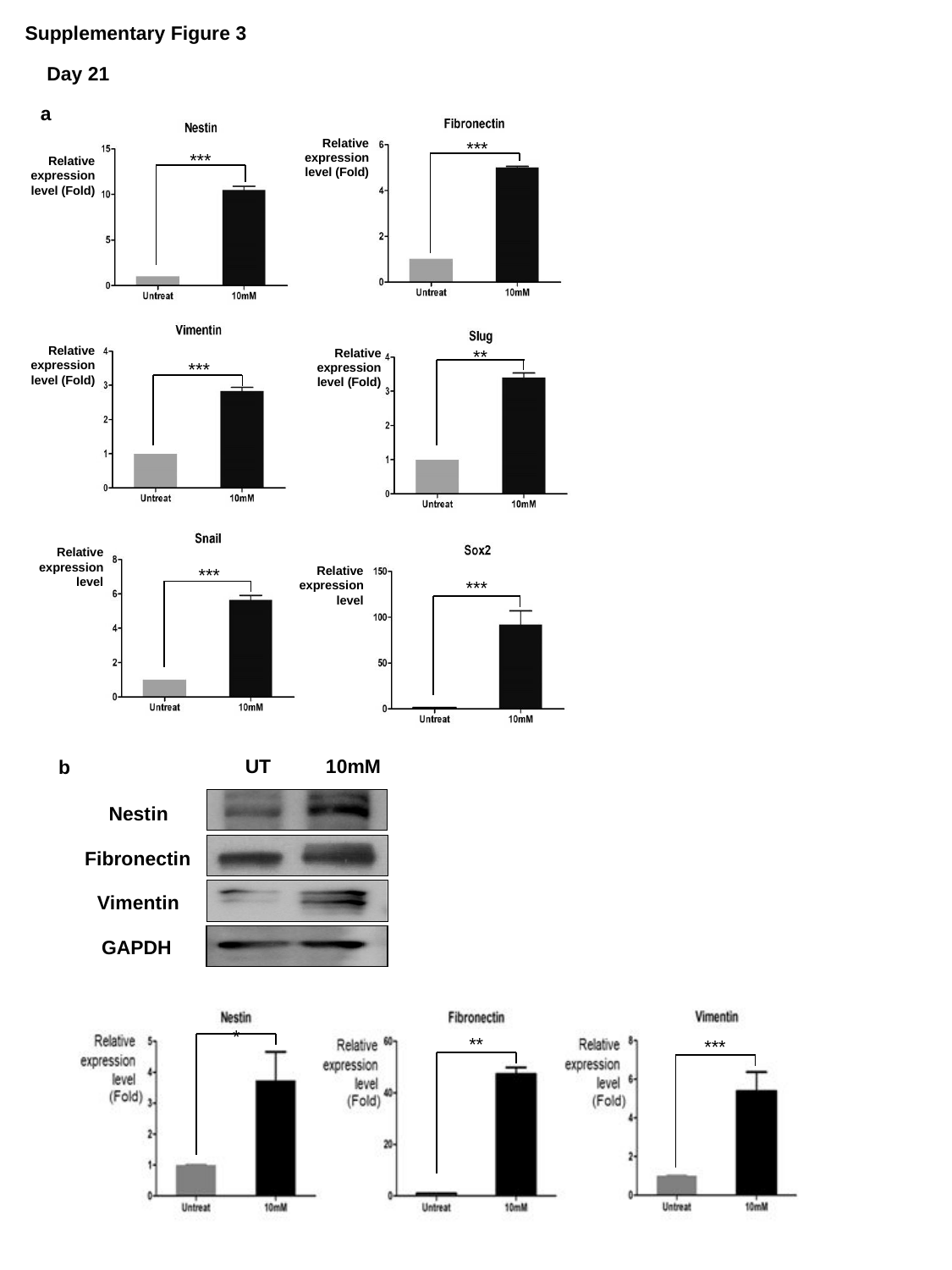

Supplementary Figure 3
Day 21
a
Relative expression
 level (Fold)
***
***
Relative expression
 level (Fold)
Relative expression
 level (Fold)
Relative expression
 level (Fold)
**
***
Relative expression
 level
Relative expression
 level
***
***
b
 UT 10mM
Nestin
Fibronectin
Vimentin
GAPDH
*
**
***

## Slide 4
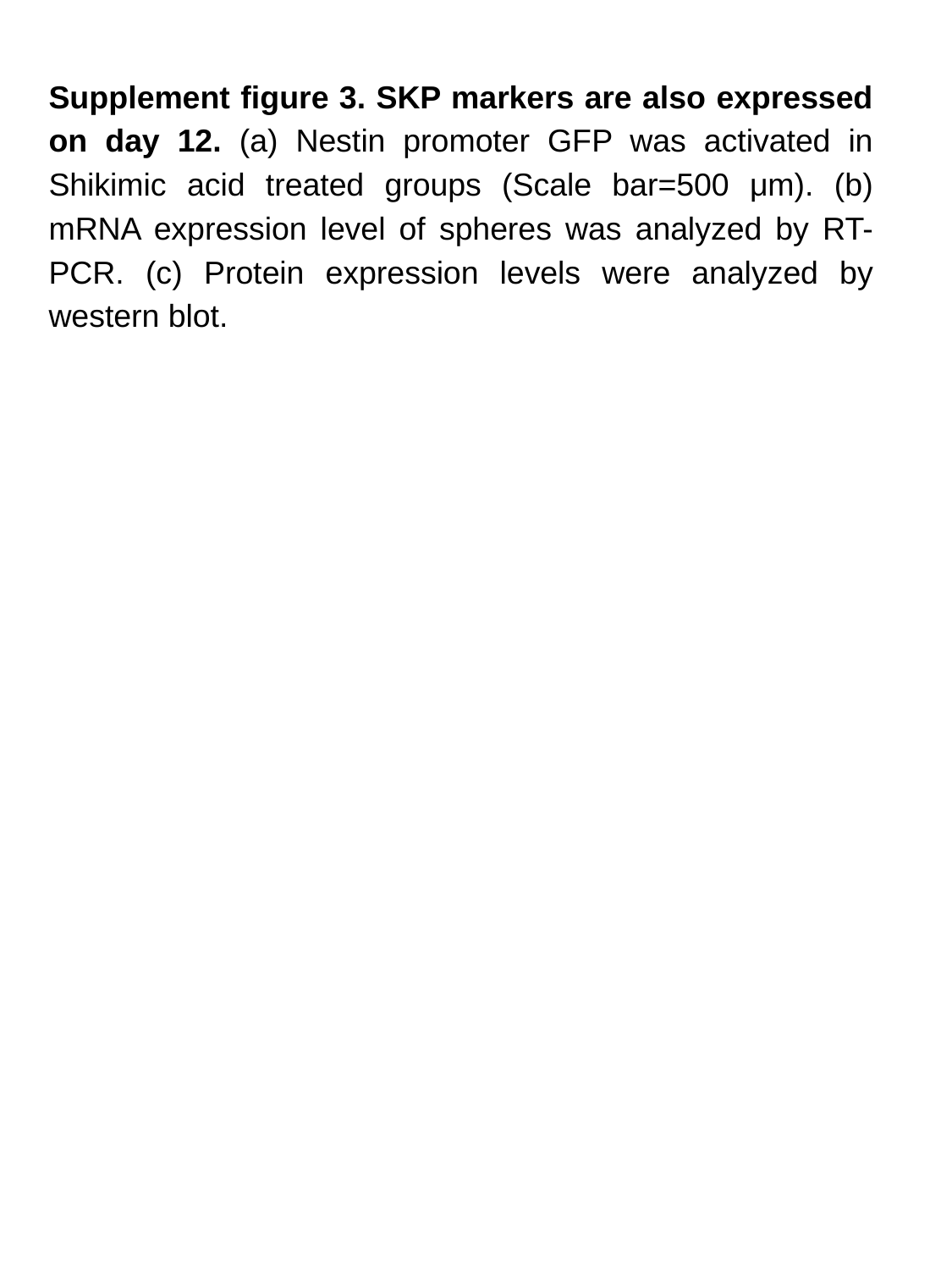

Supplement figure 3. SKP markers are also expressed on day 12. (a) Nestin promoter GFP was activated in Shikimic acid treated groups (Scale bar=500 μm). (b) mRNA expression level of spheres was analyzed by RT-PCR. (c) Protein expression levels were analyzed by western blot.

## Slide 5
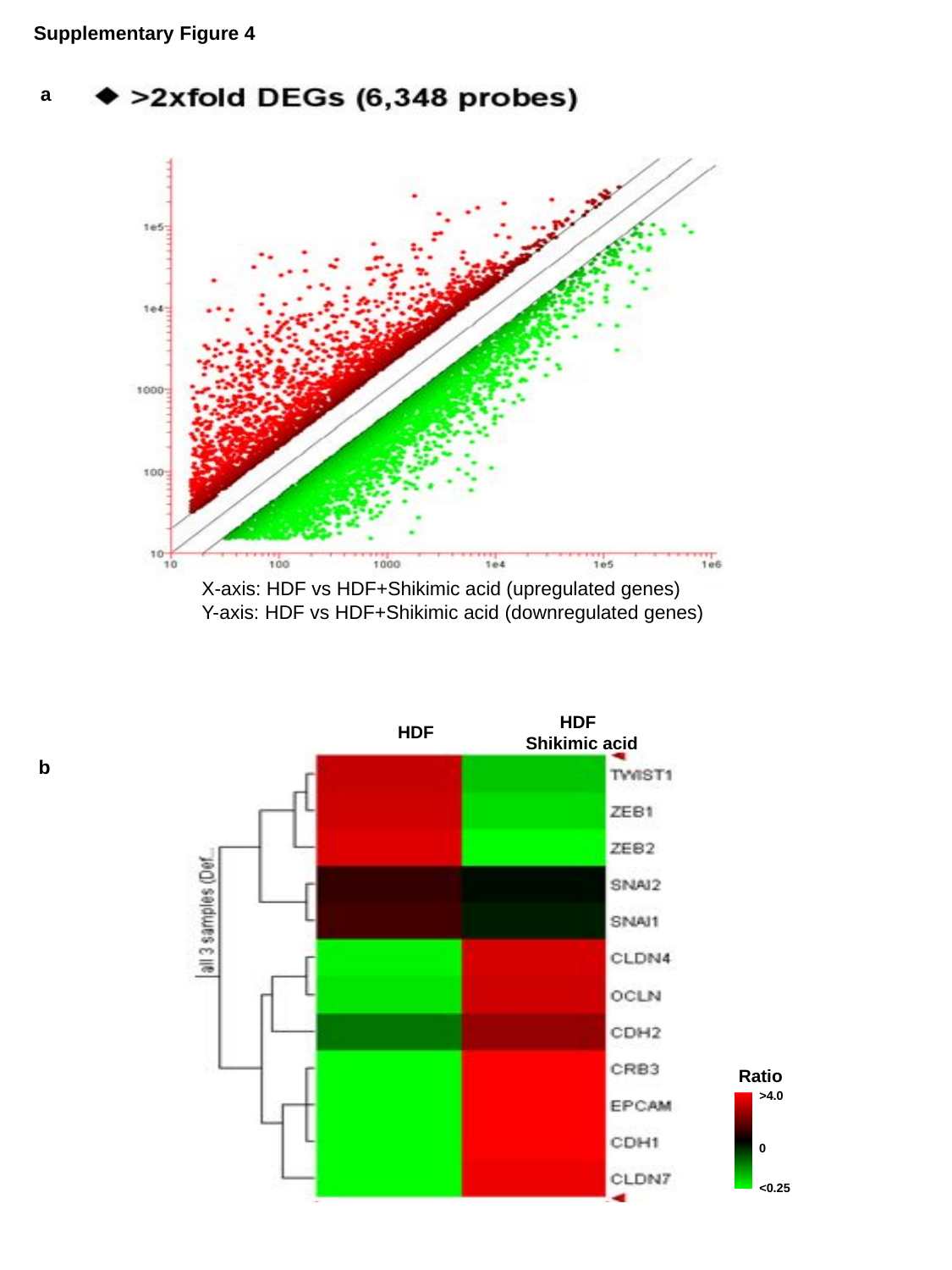

Supplementary Figure 4
a
X-axis: HDF vs HDF+Shikimic acid (upregulated genes)
Y-axis: HDF vs HDF+Shikimic acid (downregulated genes)
 HDF
Shikimic acid
HDF
b
Ratio
>4.0
0
<0.25

## Slide 6
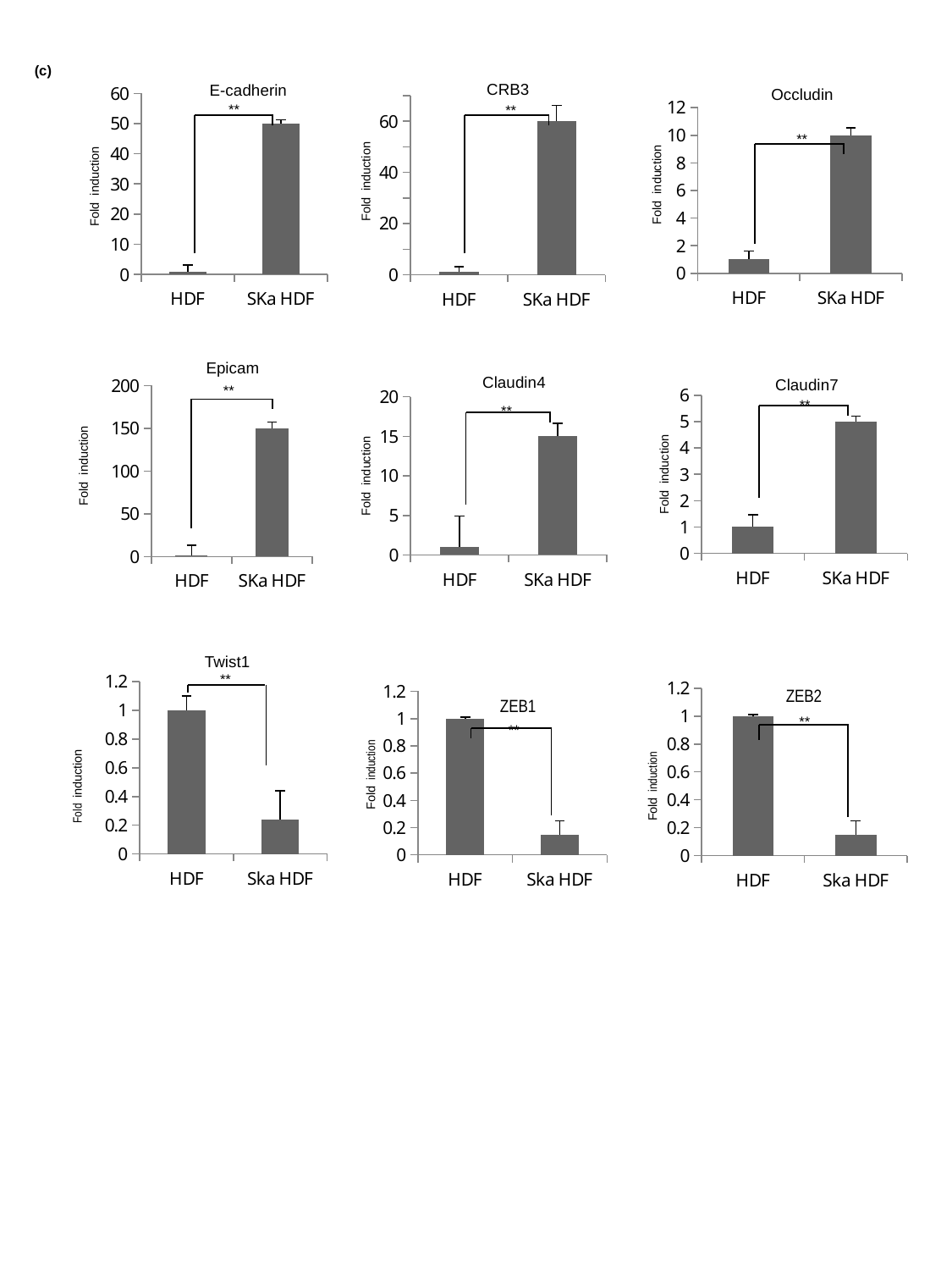

(c)
CRB3
E-cadherin
### Chart
| Category | |
|---|---|
| HDF | 1.0 |
| SKa HDF | 50.0 |Occludin
### Chart
| Category | |
|---|---|
| HDF | 1.0 |
| SKa HDF | 60.0 |
### Chart
| Category | |
|---|---|
| HDF | 1.0 |
| SKa HDF | 10.0 |**
**
**
Fold induction
Fold induction
Fold induction
Epicam
### Chart
| Category | |
|---|---|
| HDF | 1.0 |
| SKa HDF | 150.0 |
EpCAM
Claudin4
Claudin7
**
### Chart
| Category | |
|---|---|
| HDF | 1.0 |
| SKa HDF | 5.0 |
### Chart
| Category | |
|---|---|
| HDF | 1.0 |
| SKa HDF | 15.0 |**
**
Fold induction
Fold induction
Fold induction
Twist1
**
### Chart
| Category | |
|---|---|
| HDF | 1.0 |
| Ska HDF | 0.24000000000000002 |TWIST1
Fold induction
### Chart
| Category | |
|---|---|
| HDF | 1.0 |
| Ska HDF | 0.15000000000000002 |ZEB2
Fold induction
### Chart
| Category | |
|---|---|
| HDF | 1.0 |
| Ska HDF | 0.15000000000000002 |ZEB1
Fold induction
**
**

## Slide 7
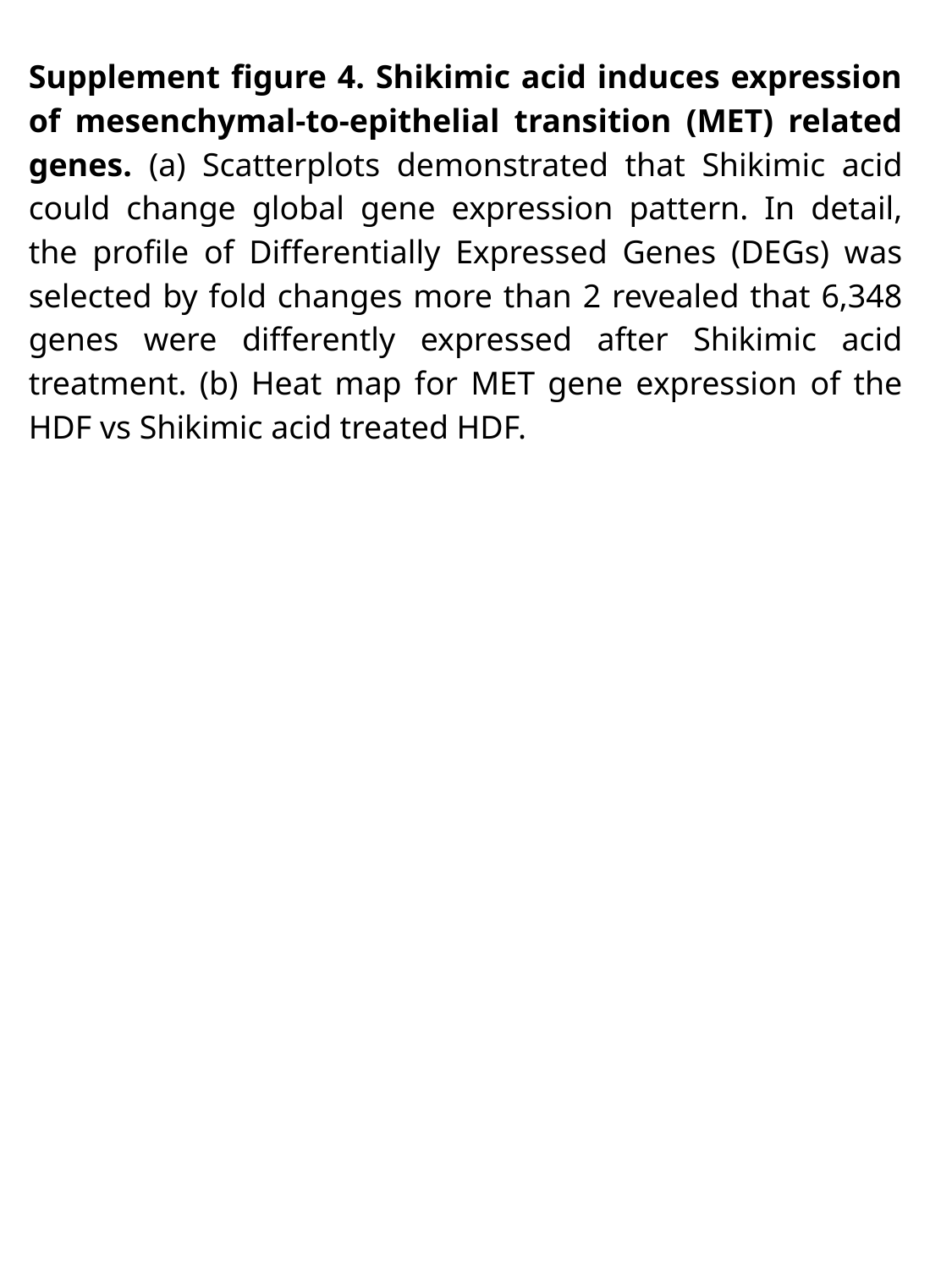

Supplement figure 4. Shikimic acid induces expression of mesenchymal-to-epithelial transition (MET) related genes. (a) Scatterplots demonstrated that Shikimic acid could change global gene expression pattern. In detail, the profile of Differentially Expressed Genes (DEGs) was selected by fold changes more than 2 revealed that 6,348 genes were differently expressed after Shikimic acid treatment. (b) Heat map for MET gene expression of the HDF vs Shikimic acid treated HDF.

## Slide 8
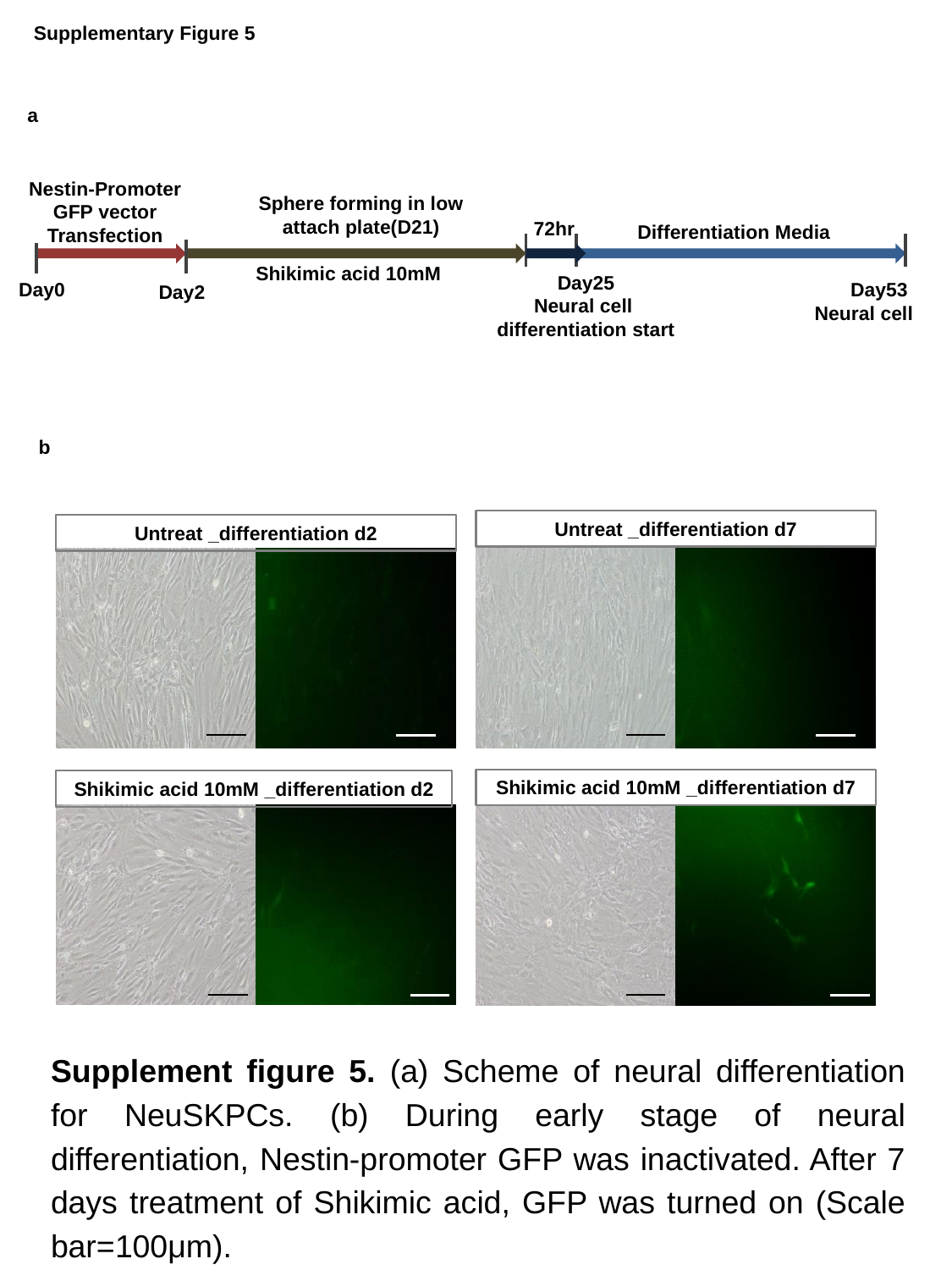

Supplementary Figure 5
a
Nestin-Promoter GFP vector Transfection
Sphere forming in low attach plate(D21)
72hr
Differentiation Media
Shikimic acid 10mM
Day25
Neural cell
differentiation start
Day0
Day53
Neural cell
Day2
b
Untreat _differentiation d7
Untreat _differentiation d2
Shikimic acid 10mM _differentiation d7
Shikimic acid 10mM _differentiation d2
Supplement figure 5. (a) Scheme of neural differentiation for NeuSKPCs. (b) During early stage of neural differentiation, Nestin-promoter GFP was inactivated. After 7 days treatment of Shikimic acid, GFP was turned on (Scale bar=100μm).

## Slide 9
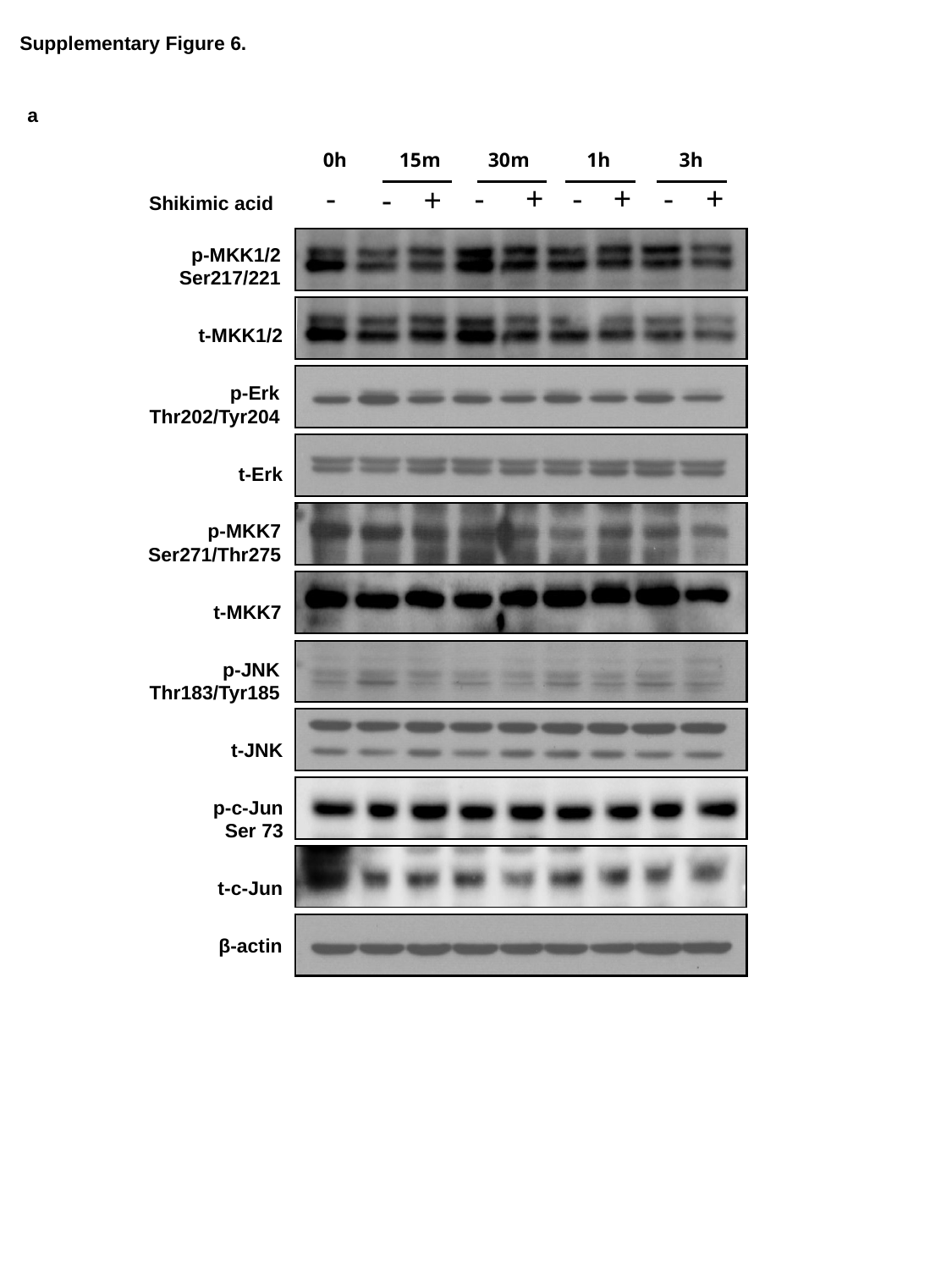

Supplementary Figure 6.
a
0h
15m
30m
1h
3h
-
-
+
-
+
-
+
+
-
Shikimic acid
p-MKK1/2
Ser217/221
t-MKK1/2
p-Erk
Thr202/Tyr204
t-Erk
p-MKK7
Ser271/Thr275
t-MKK7
p-JNK
Thr183/Tyr185
t-JNK
p-c-Jun
Ser 73
t-c-Jun
β-actin

## Slide 10
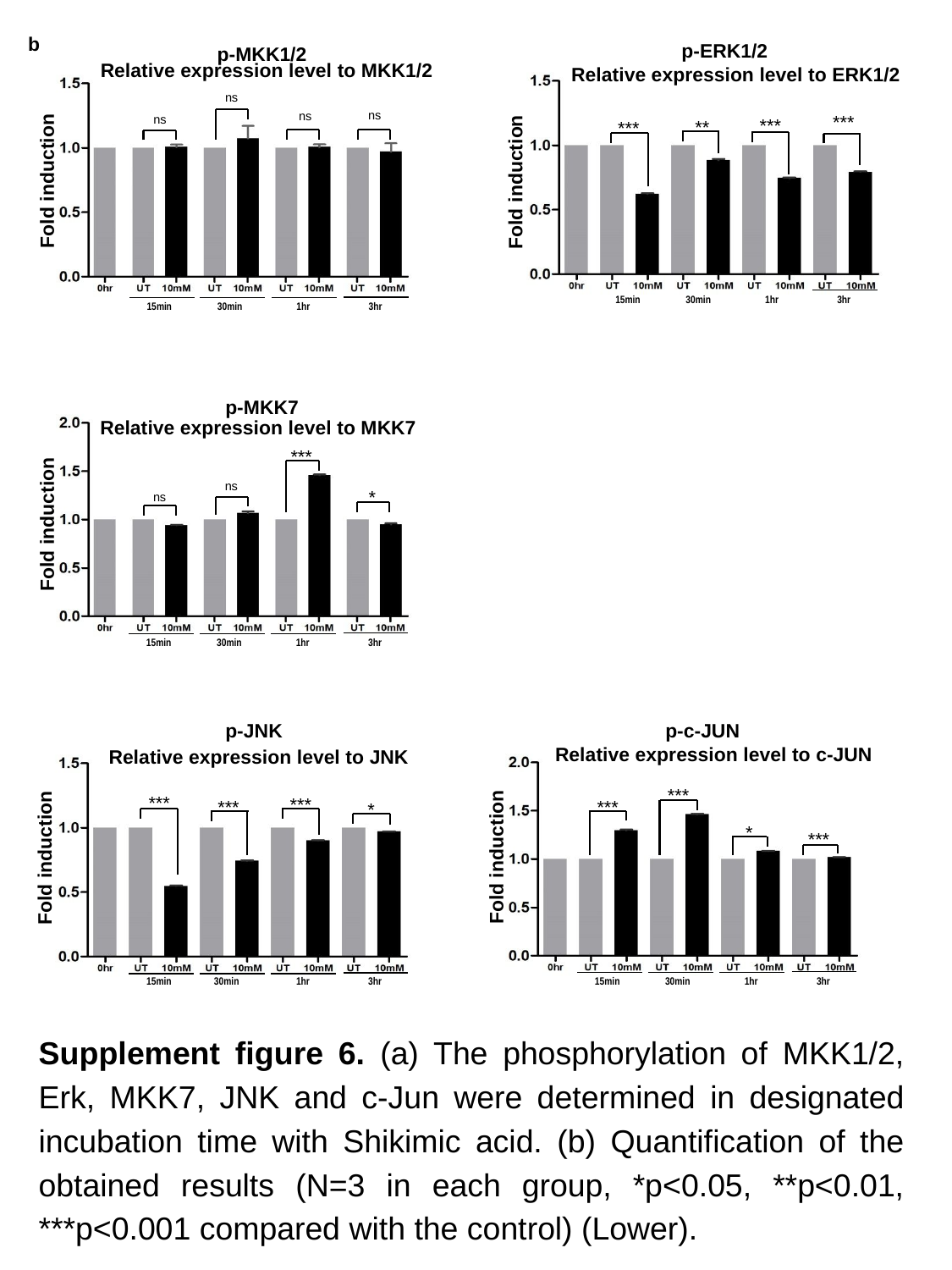

b
p-ERK1/2
p-MKK1/2
Relative expression level to MKK1/2
Relative expression level to ERK1/2
ns
ns
ns
***
ns
***
**
***
Fold induction
Fold induction
15min
30min
1hr
3hr
15min
30min
1hr
3hr
p-MKK7
Relative expression level to MKK7
***
ns
*
ns
Fold induction
15min
30min
1hr
3hr
p-JNK
p-c-JUN
Relative expression level to c-JUN
Relative expression level to JNK
***
***
***
***
***
*
*
***
Fold induction
Fold induction
15min
30min
1hr
3hr
15min
30min
1hr
3hr
Supplement figure 6. (a) The phosphorylation of MKK1/2, Erk, MKK7, JNK and c-Jun were determined in designated incubation time with Shikimic acid. (b) Quantification of the obtained results (N=3 in each group, *p<0.05, **p<0.01, ***p<0.001 compared with the control) (Lower).

## Slide 11
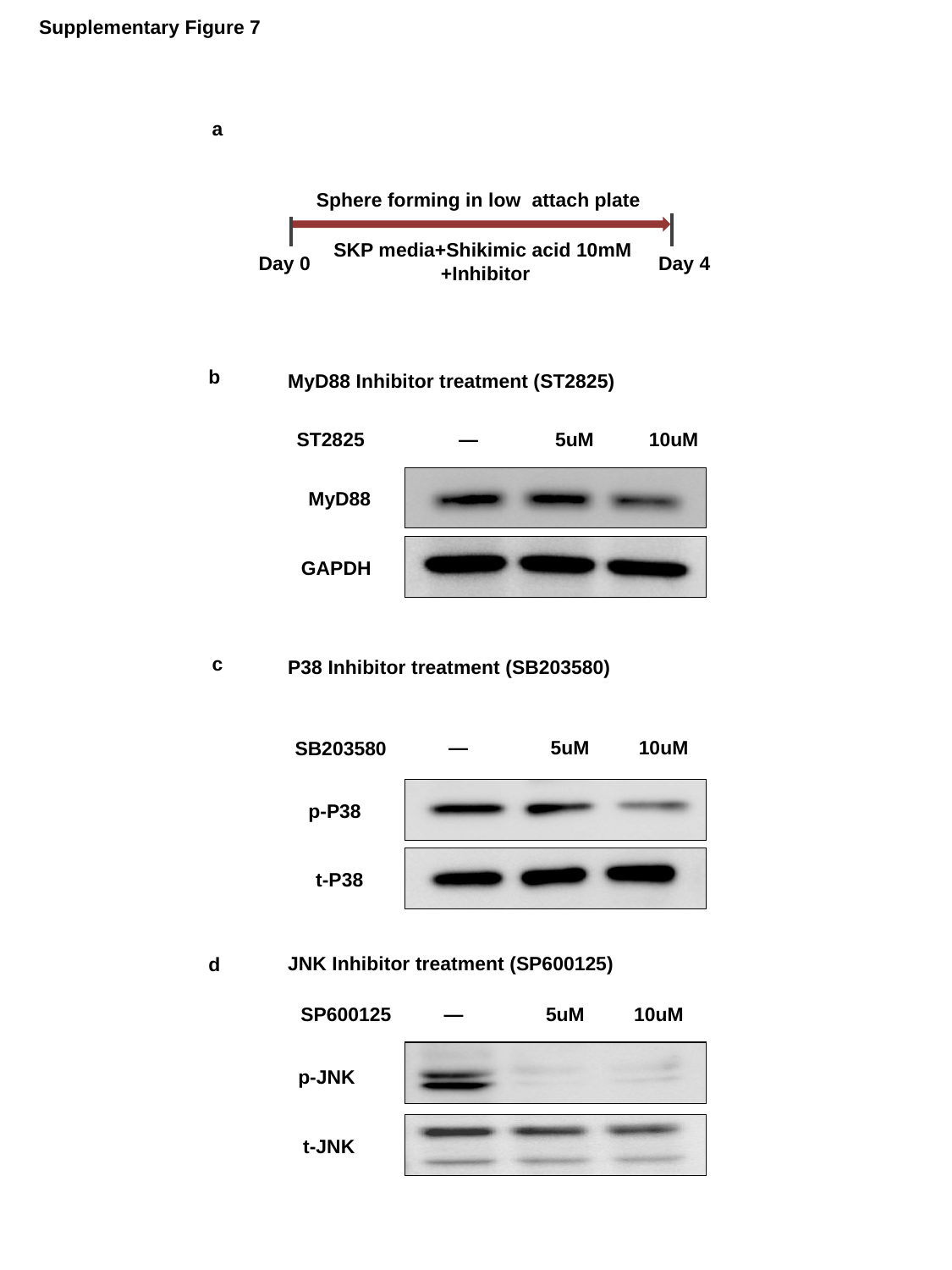

Supplementary Figure 7
a
Sphere forming in low attach plate
SKP media+Shikimic acid 10mM
+Inhibitor
Day 4
Day 0
b
MyD88 Inhibitor treatment (ST2825)
ST2825
— 5uM 10uM
MyD88
GAPDH
c
P38 Inhibitor treatment (SB203580)
SB203580
— 5uM 10uM
p-P38
t-P38
d
JNK Inhibitor treatment (SP600125)
SP600125
— 5uM 10uM
p-JNK
t-JNK

## Slide 12
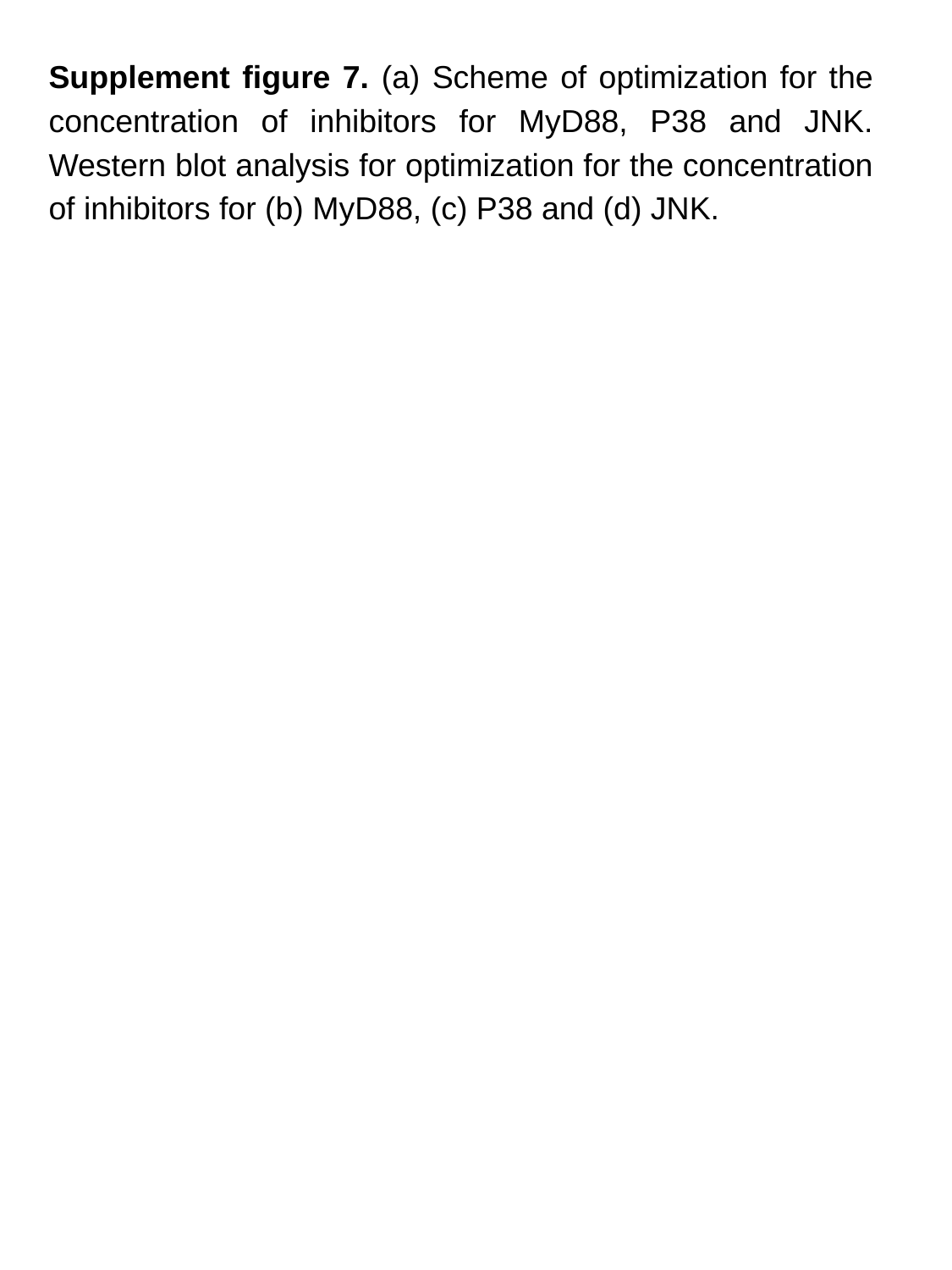

Supplement figure 7. (a) Scheme of optimization for the concentration of inhibitors for MyD88, P38 and JNK. Western blot analysis for optimization for the concentration of inhibitors for (b) MyD88, (c) P38 and (d) JNK.

## Slide 13
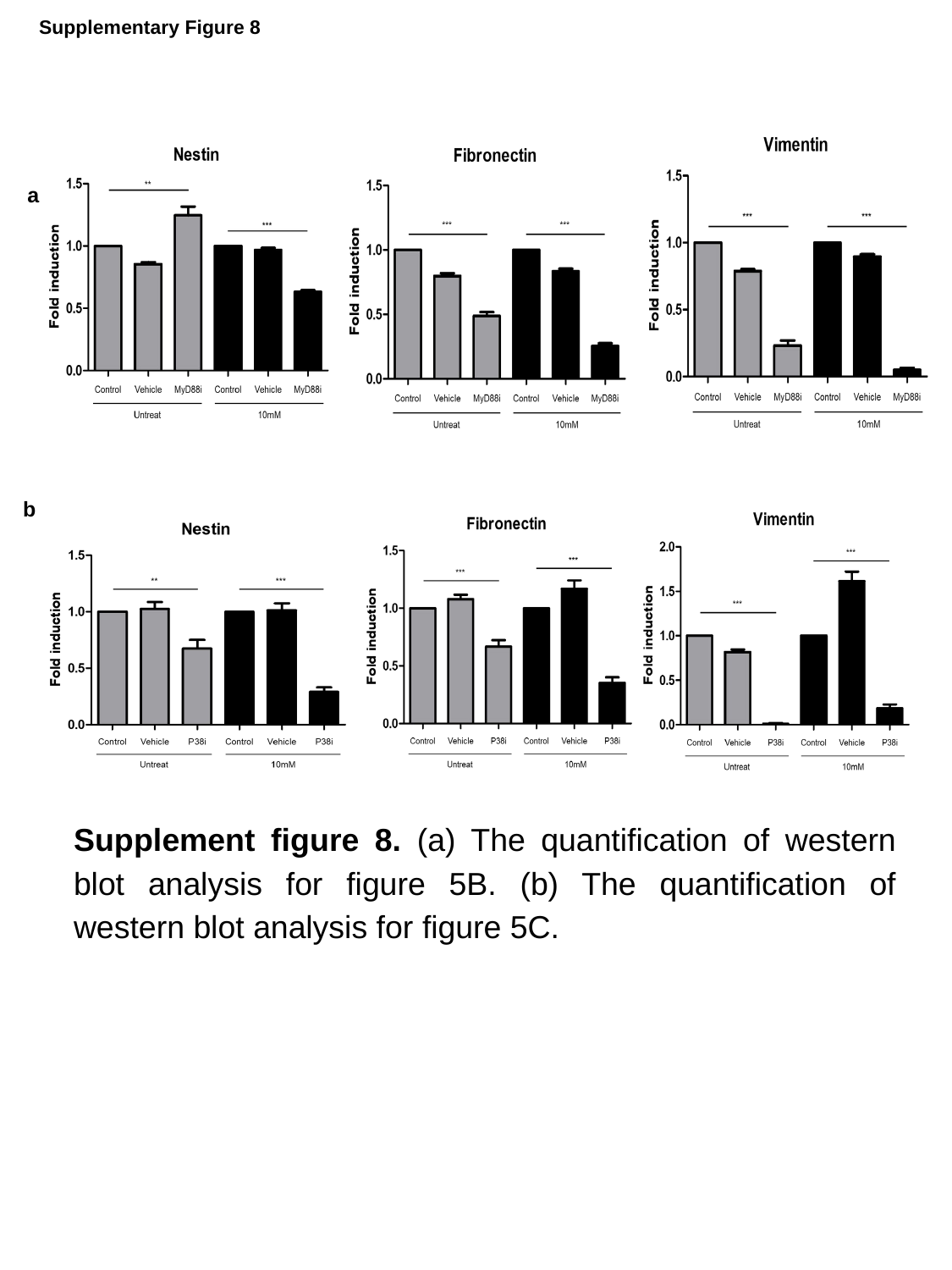

Supplementary Figure 8
a
b
Supplement figure 8. (a) The quantification of western blot analysis for figure 5B. (b) The quantification of western blot analysis for figure 5C.

## Slide 14
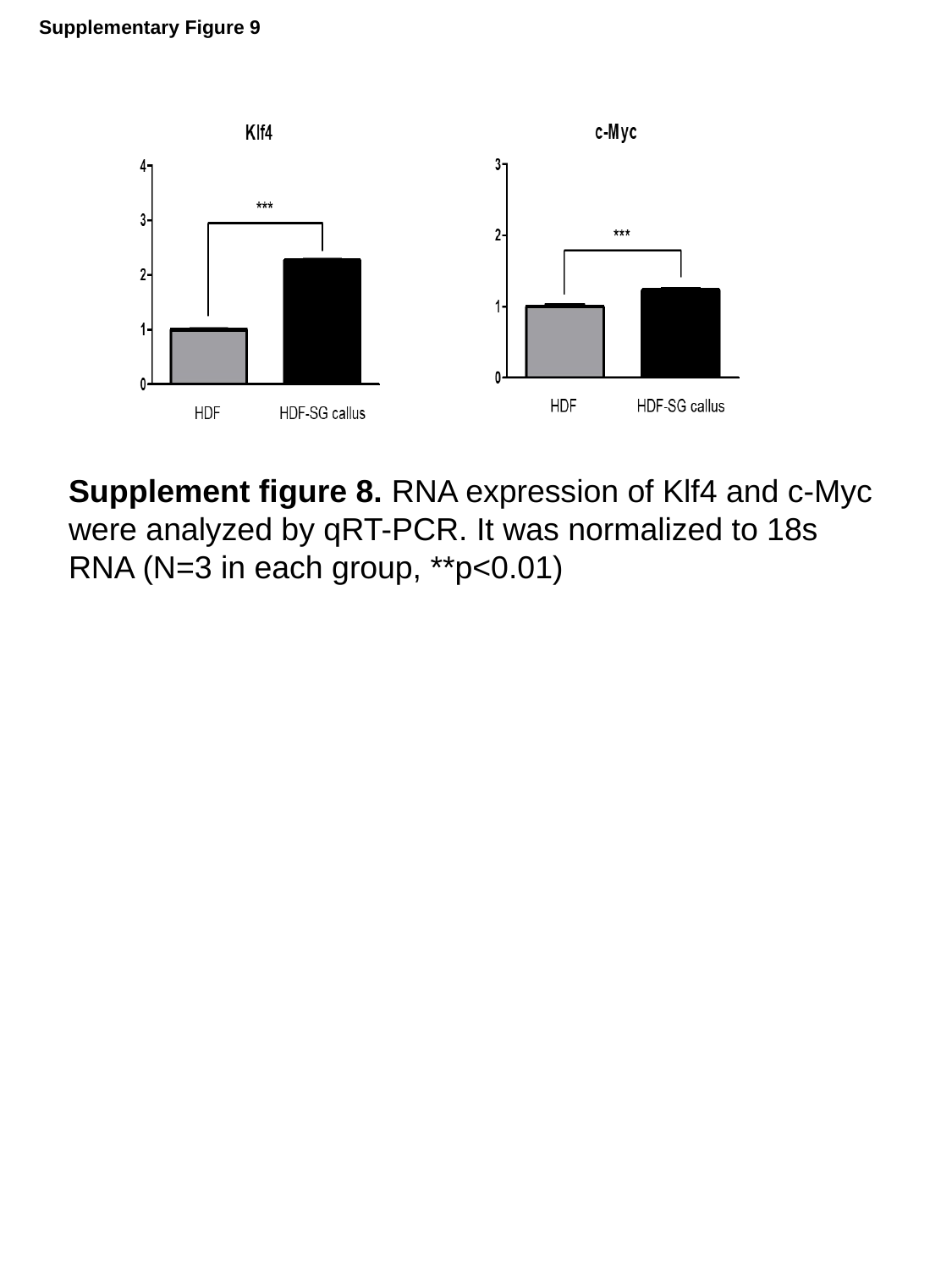

Supplementary Figure 9
Supplement figure 8. RNA expression of Klf4 and c-Myc were analyzed by qRT-PCR. It was normalized to 18s RNA (N=3 in each group, **p<0.01)
