# Supplementary material for: Plant callus-derived shikimic acid regenerates human skin through converting human dermal fibroblasts into multipotent skin-derived precursor cells
Source: Stem Cell Res Ther. 2021 Jun 11;12:346. doi: 10.1186/s13287-021-02409-3 (PMC8196440; doi:10.1186/s13287-021-02409-3)
Supplement: Supplementary file 2 — Additional file 2: Table S1-2. [file 13287_2021_2409_MOESM2_ESM.docx]

**Table S1**

| **Gene name** | **F,R** | **Sequence (5’→3’)** | **Tm (℃)** |
| --- | --- | --- | --- |
| **Nestin** | **F** | **GTAGCTCCCAGAGAGGGAA** | **60** |
|  | **R** | **CTCTAGAGGGCCAGGGACTT** | **60** |
| **Fibronectin** | **F** | **CCGCCGAATGTAGGACAAGAA** | **60** |
|  | **R** | **CTGTCAGAGTGGCACTGGTA** | **59** |
| **Vimentin** | **F** | **GGACCAGCTAACCAACGACA** | **60** |
|  | **R** | **AAGGTCAAGACGTGCCAGAG** | **60** |
| **Oct4** | **F** | **GAAGGAGAAGCTGGAGCAAAAC** | **60** |
|  | **R** | **CCCACATCGGCCTGTGTAT** | **60** |
| **Sox2** | **F** | **GGACAGTTACGCGCACATGA** | **61** |
|  | **R** | **AGCCGTTCATGTAGGTCTGC** | **69** |
| **Nanog** | **F** | **GGCTCTGTTTTGCTATATCCCCTAA** | **60** |
|  | **R** | **CATTACGATGCAGCAAATACAAGA** | **58** |
| **TERT** | **F** | **AAACCTTCCTCAGCTATGCCC** | **60** |
|  | **R** | **CCGCAAGAACCCCAAAGAGTT** | **61** |
| **C/EBPα** | **F** | **TATAGGCTGGGCTTCCCCTT** | **60** |
|  | **R** | **AGCTTTCTGGTGTGACTCGG** | **60** |
| **PPARγ** | **F** | **GCTGACCAAAGCAAAGGCG** | **60** |
|  | **R** | **GCCCTGAAAGATGCGGATG** | **59** |
| **FABP4** | **F** | **CCTTAGATGGGGGTGTCCTG** | **59** |
|  | **R** | **TCGTGGAAGTGACGCCTTTC** | **60** |
| **Runx2** | **F** | **CACATGATTCTGCCTCTCCAGT** | **60** |
|  | **R** | **TGTGGTTGTTTGTGAGGCGA** | **60** |
| **Osterix** | **F** | **GACCTCCAGAGAGGAGAGACTCG** | **62** |
|  | **R** | **CAAGGAGCCAGGCAGATGGAG** | **62** |
| **Osteocalcin** | **F** | **TCCTTTGGGGTTTGGCCTAC** | **60** |
|  | **R** | **CCAGCCTCCAGCACTGTTTA** | **60** |
| **Osteopontin** | **F** | **TCCCTGTGTTGGTGGAGGAT** | **60** |
|  | **R** | **GTTTTCCTTGGTCGGCGTTT** | **59** |
| **ALP** | **F** | **CTATCCTGGCTCCGTGCTCC** | **62** |
|  | **R** | **AGATGCAATCGACGTGGGTG** | **60** |
| **p-CREB** | **F** | **AATCGGATCTGATGGTGGTGG** | **62** |
|  | **R** | **AGCGGCTCGCAGAGCT** | **62** |
| **GAPDH** | **F** | **AAGGTCGGAGTCAACGGATTT** | **60** |
|  | **R** | **GTTCTCAGCCTTGACGGTGC** | **60** |
| **18S rRNA** | **F** | **GGCCCTGTAATTGGAATGAGTC** | **59** |
|  | **R** | **CCAAGATCCAACACGAGCTT** | **58** |

**Table S2**

|  | **Sequence Product** | **size** | **Tm(℃)** |
| --- | --- | --- | --- |
| **p-CREB_Foward** | **AGGCTGGAACGTCCTG** | **383bp** | **62℃** |
| **p-CREB_Reverse** | **AGCGGCTCGCAGAGCT** |  | **62℃** |
