# Supplementary material for: Plant callus-derived shikimic acid regenerates human skin through converting human dermal fibroblasts into multipotent skin-derived precursor cells
Source: Stem Cell Res Ther. 2021 Jun 11;12:346. doi: 10.1186/s13287-021-02409-3 (PMC8196440; doi:10.1186/s13287-021-02409-3)
Supplement: Supplementary file 3 — Additional file 3. [file 13287_2021_2409_MOESM3_ESM.docx]

**HPLC analysis**

HPLC analysis of SG callus extract were carried out on a Waters 1525 m Binary HPLC Pump (Waters) and photodiode array detector (Waters). Separations were performed on: Gemini 5 m C18 110A (5 mm, 4.6×250 nm, Phenomenex). Gradient elution of the samples and standard were performed using 0.1 % TFA water solution (eluent A) and mixture of acetonitrile (eluent B, 30:70, v/v). The flow rate was 1 ml/min and detected at the UV wavelength of 230 nm. The SG Callus extract was dissolved in Butylene Glycerol and ethanol mixture (70;30, v/v) at a concentration of 10 % SG Callus extract. The retention time of each component was showed at different time points.

**plant callus preparation**

The seeds of Sequoiadendron Giganteum were submerged in 70% ethanol for 30 seconds and shake in 0.3% Sodium hypochlorite for 10-15 min. They were washed with distilled water several times. The young stem and leaves of Sequoiadendron Giganteum cultured for 10 days after sprouting. Then, they were harvested and segmented. The plant segments (0.5 cm) were inoculated onto basal media supplemented with 10 mg/ml IBA (Indole-3-butyric acid), 0.02 mg/L Kinetin, 30 g/L sucrose, 2.0 g/L Gelrite (Sigma). The plant segments were incubated at 25 ℃ to induce plant callus. Propagated Sequoiadendron Giganteum plant callus were dehydrated with paper tissue and dried at 60 ℃ for 2 days. The dried callus powder (100 g) was rotary extracted with 1 L distilled water at 65 ℃ for 2 hours**.**

**In vitro differentiation of NeuSKPCs and immunocytochemistry**

For adipogenic differentiation, NeuSKPCs were attached 0.1% gelatin coated cultured plate and initiated differentiation 3 days later in order to wait until cells were stretched out from sphere. Adipogenic differentiation media contains minimum essential medium (MEM;Gibco), 15% Rabbit serum (Sigma Aldrich), 0.1 mM dexamethasone, 0.5 nM isobuthylxanthine (all from sigma) and 10 mg/ml insulin. The media was changed every 3 days for 14 days. For histological evaluation of differentiated adipocytes, cells were stained using Oil Red O. Osteogenic differentiation media consist of MEM (Gibco), 10 % FBS (Gibco), 10 mM b-glycerol phosphate, 0.1 mM dexamethasone, 50 mM ascorbic acid (all from Sigma). The medium was changed every 3 days for 28 days. Osteogenically differentiated cells were confirmed using von Kossa staining. For neural cell differentiation, NeuSKPCs were attached on 0.02 mg/ml of laminin and 0.2 mg/ml of poly-D-lysine coated plate. Differentiation was initiated 5 day after the sphere attachment. DMEM:F12(3:1) (Gibco) supplemented with 1 % FBS (Gibco), 10 ng/ml Neutrophilin-3, 50 ng/ml Nerve growth factor and 50 ng/ml Brain-derived neurotrophic factor (all from Peprotech) for 5 weeks. Differentiated neural cells were stained using MAP2, Tuj1, NeuN (all stained for 1:200, from Santa-cruz biotech).

**Chromatin immunoprecipitation (ChIP)**

Chromatin immunoprecipitation (ChIP assay) was performed using Chromatin Immuno-precipitation kit (Merk Millipore) following the manufacturer’s guide. NeuSKPCs were fixed in 1 % of formaldehyde for 10 minutes in 37 ℃. Cells were lysed using SDS Lysis buffer (Merk Millipore) and incubated on ice for 10 min. After the lysis, the cross-linked DNA was sheared to 200-1000bp length by sonication in Bioruptor. Sonicated chromatins were diluted with ChIP dilution buffer (Merk Millipore) and incubated overnight at 4 ℃ with primary antibody (phospho-CREB (9198S), phospho-c-Fos (3270S) (Cell signaling)) or normal rabbit IgG (Cell signaling) as a negative control. Protein A agarose beads were added to collect antibody-chromatin complex. Beads were washed in several buffers (Low-salt buffer, High-salt buffer, LiCl buffer and TE buffer) and protein-DNA complex was eluted and reverse cross linked with elution buffer for overnight, 65 ℃. DNA purification was performed using QIAquick PCR Purification kit (Qiagen). Purified DNA was amplified the target sequence by PCR. Primer sequences used to amplify promoter region were listed on table s2. Relative binding was measured as the band intensity of the Chip sample for the input sample.

**Histology and Immunohistochemistry**

For immunohistochemistry, antigen retrieval was performed by steaming tissue sections in citrate buffer for 15 min and immunostaining was performed with antibodies antigen type 1 collagen (Santa cruz biotechnology) and Cytokeratin 10 (Santa cruz biotechnology). Staining localization was detected using Alexa Fluor 488-conjugated goat anti-mouse secondary antibody (Invitrogen). The tissues were counterstained with 1 mg/mL DAPI (4’,6’-diamidino-2-phenylindole), diluted in the blocking buffer for 1 min. A Z-series (0.2 mm steps) of optical sections was digitally imaged on an LSM 510 META laser scanning microscope (Carl Zeiss, Oberkochen, Germany).
